# How can we improve latent tuberculosis infection management using behaviour change wheel: a systematic review

**DOI:** 10.1093/pubmed/fdad051

**Published:** 2023-05-05

**Authors:** Yen Jun Wong, Khuen Yen Ng, Shaun Wen Huey Lee

**Affiliations:** School of Pharmacy, Monash University Malaysia, Jalan Lagoon Selatan, 47500 Subang Jaya, Selangor, Malaysia; School of Pharmacy, Monash University Malaysia, Jalan Lagoon Selatan, 47500 Subang Jaya, Selangor, Malaysia; School of Pharmacy, Monash University Malaysia, Jalan Lagoon Selatan, 47500 Subang Jaya, Selangor, Malaysia; School of Pharmacy, Taylor’s University Lakeside Campus, Jalan Taylors, 47500 Subang Jaya, Selangor, Malaysia; Center of Global Health, Perelman School of Medicine, University of Pennsylvania, Philadelphia, PA 19104, USA

**Keywords:** behaviour, systematic review, tuberculosis

## Abstract

**Background:**

To ensure the effective delivery of latent tuberculosis infection (LTBI) care, it is vital to overcome potential challenges in LTBI management. This systematic review aims to identify the barriers and interventions to improve LTBI management using the Capability, Opportunity, and Motivation-Behaviour (COM-B) model and Behaviour Change Wheel (BCW).

**Methods:**

A systematic literature search was performed on five electronic databases from database inception to 3 November 2021. A two-step technique was used in the data synthesis process: (i) the barriers of LTBI management were identified using the COM-B model, followed by (ii) mapping of intervention functions from BCW to address the identified barriers.

**Results:**

Forty-seven eligible articles were included in this review. The findings highlighted the need for a multifaceted approach in tackling the barriers in LTBI management across the public, provider and system levels. The barriers were summarized into suboptimal knowledge and misperception of LTBI, as well as stigma and psychosocial burden, which could be overcome with a combination of intervention functions, targeting education, environment restructuring, persuasion, modelling, training, incentivization and enablement.

**Conclusions:**

The remedial strategies using BCW to facilitate policy reforms in LTBI management could serve as a value-added initiative in the global tuberculosis control and prevention program.

## Introduction

Tuberculosis (TB) is one of the leading causes of mortality worldwide, affecting approximately 10.6 million of the global population, with more than 1.6 million death cases reported in 2021.^1^ As such, early diagnosis and treatment of TB cases by knowledgeable and skilled healthcare providers are key in addressing this global health issue. To strengthen TB service delivery, the Capability, Opportunity, and Motivation-Behaviour (COM-B) model has been applied to identify and address the barriers in TB management, particularly in improving TB case detection.^2^^,^^3^ This model has been used with the behaviour change wheel (BCW) to identify intervention functions and policy categories, which facilitate improvement in health delivery and services.^4^

In response, the World Health Organization (WHO) has taken the lead to accelerate global effort in TB eradication through the WHO END TB Strategy.^5^ In addition to active TB case finding and treatment, it also advocates the prioritization of latent tuberculosis infection (LTBI) management as LTBI could be the reservoir for future TB cases.^5^^,^^6^ Therefore, prevention of TB reactivation through LTBI treatment and management in specific high-risk populations is one of the milestones proposed at the United Nations High-Level Meeting.^1^ One issue identified in LTBI management was the lack of understanding and awareness of LTBI testing and treatment among healthcare providers and the general public, particularly populations from countries where TB is common.^7^^,^^8^

However, unlike TB, a collective presentation on the knowledge, attitude and practice (KAP) of LTBI has not been conducted to study the barriers and facilitators in LTBI care. Similar to TB, the COM-B model can be a useful guide to identify the barriers in LTBI management from the KAP studies, followed by mapping of intervention functions from BCW to address the identified barriers.

It is essential to pinpoint and address the gaps in LTBI management. This paper aims to (1) identify the barriers in LTBI management from KAP studies using the COM-B model and to (2) identify intervention functions guided by BCW, as strategies to improve LTBI management.

## Methods

This systematic review was reported according to the Preferred Reporting Items for Systematic Reviews and Meta-Analyses 2020 (Supplementary Appendix 1).^9^ The review protocol is registered with PROSPERO (Registration ID CRD42020166667).

### Eligibility criteria

Observational studies reporting KAP outcomes on LTBI irrespective of study setting or publication year were selected for screening and review. We limited our search to studies published in English. Details on the inclusion and exclusion criteria are described in Supplementary Appendix 2.

### Search strategy

Searches were performed on five electronic databases: PubMed, EMBASE, CINAHL-Plus, Web of Science and ProQuest, from database inception until 3 November 2021. This was supplemented by citation search of identified literature.

Medical subject headings (MeSH) terms and keywords which described health care workers or general public, LTBI, KAP, were used to generate lists of publications for screening. The full search terms can be found in Supplementary Appendix 3.

### Study selection and data extraction

Two reviewers (YJW and SWHL) independently screened titles and abstracts of references. Full texts of identified studies were independently assessed and reviewed in accordance with the eligibility criteria. Reasons for excluded studies are found in Supplementary Appendix 4.

Data were extracted using a predeveloped data collection form. Extracted data included: first author, year of publication, location of the study, study population, questionnaire structure, KAP category, survey questions with the corresponding responses by one reviewer (YJW). The findings were cross-checked by second reviewer (SWHL), with disagreement resolved by discussion or adjudication by a third reviewer (KYN).

We contacted authors from four studies to request the survey instruments used in their studies, to facilitate our study selection process.^10–13^ All authors responded to our request.

### Risk of bias evaluation

As there were no standardized or validated tools to evaluate the risk of bias in KAP survey,^14^^,^^15^ we adapted the checklist developed by Hoy and colleagues since it was consistent with the screening criteria for KAP survey proposed by Agarwal and colleagues (Supplementary Appendix 5).^14^^,^^16^

### Data synthesis

A three-step process was applied for data synthesis. First, we extracted and coded each question from the KAP questionnaire as described previously where they were categorized as either knowledge, attitude or practice. We then identified the target audience, which the questionnaires were administered to, i.e. general public, provider or system. These were further sorted into the COM-B model domains: (i) physical capability, (ii) psychological capability, (iii) automatic motivation, (iv) reflective motivation, (v) physical opportunity or (vi) social opportunity. We subsequently mapped these domains to the BCW to determine which intervention function could be used to address the identified barriers. All data analysis was performed in Microsoft Excel (Richmond, USA).

## Results

### Study characteristics

**Fig. 1:**
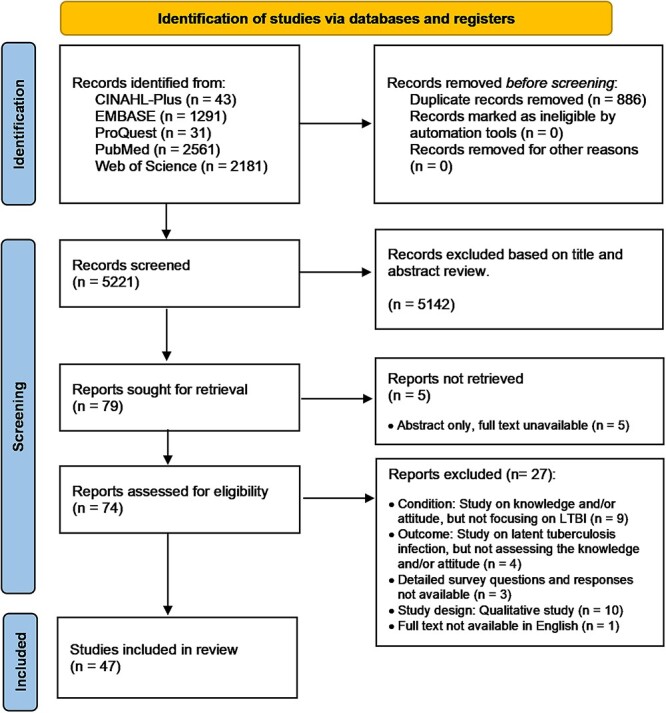
PRISMA flow diagram showing the selection of included studies.

The literature search identified 5221 studies for evaluation, 74 were screened for inclusion and 47 eligible studies were included in this study (Fig. 1).^10^^,^^17–62^

These studies had invited a total of 35 694 participants, with 22 991 responses, of which 22 802 were fully completed. The response rates for each survey varied, ranging from 72.7% to as high as 100% full completion. Only eight out of 47 studies were conducted in countries that are listed in the WHO Global High Burden Country lists 2021–2025,^1^ namely Brazil (*n* = 2), China (*n* = 2), India (*n* = 1), Peru (*n* = 1), South Africa (*n* = 1) and Thailand (*n* = 1) (Table 1 and Supplementary Appendix 6).^24^^,^^29^^,^^41^^,^^45^^,^^50^^,^^52^^,^^56^^,^^62^

**Table 1:** Study characteristics of the included studies

Study	Study location	Study population	Sample size	Survey completion rate (%)	Data collection period	Study design	Study objective
Ailinger 2004^17^	TB clinic, in a county with a population of 20% Latino origin, the US	Adult Latino immigrants from Central America and Bolivia treated for LTBI	82	100	Not reported	Cross-sectional	To examine TB knowledge in Latino immigrants receiving LTBI therapy
Alotaibi 2019^18^	Mecca, Saudi Arabia	HCWs—Doctors and nurses serving pilgrims during Hajj	540	100	2–12 September 2016	Cross-sectional	To assess knowledge, attitude and practice of HCWs deployed during 2016 Hajj regarding TB and its management
Atchison 2015^b^^19^	England, the UK	HCWs—General practitioners in primary care; some had experience in LTBI	112	100	February to April 2014	Mixed-method	To investigate perceived barriers and enablers to primary care-based LTBI treatment among UK general practitioners (GPs).
Bhanot 2012^20^	Maimonides Medical Centre, New York, the USA	HCWs—Attending physicians and physicians in training	87^a^	≈100	Not reported	Cross-sectional	To explore the attitudes of physicians towards self-treatment of LTBI
Biedenharn 2015^21^	Southwest Ohio public health clinic, the USA	Persons eligible for LTBI treatment	42	100	1 January to 31 December 2014	Cross-sectional	To identify any knowledge, attitude or social characteristics that are associated with LTBI treatment acceptance or refusal
Butcher 2013^b^^22^	Immigrant/refugee and TB clinics, Royal Melbourne Hospital, Australia	Foreign-born individuals (immigrants and refugee) treated for LTBI	Questionnaire 1:20 Questionnaire 2:14 (out of 20 from Questionnaire 1) + 32	100	5 May to 9 October, 2009	Cross-sectional	To assess refugee and immigrant patients’ knowledge and understanding of information given about LTBI, the treatment regimen and potential side effects of isoniazid
Cantini 2016^23^	Nationwide, Italy	HCWs—Rheumatologists	393	100	January to March 2012	Cross-sectional	To investigate Italian rheumatology practice regarding LTBI detection and TB prevention in patients requiring anti-TNF therapy
Chiang 2015^24^	102 health centres in Lima Ciudad and Lima Este, Peru	HCWs—NTP physicians’, nurses’ and nursing technicians’; with experience in TB	310 (103 physicians, 106 nurses, 101 nursing technicians)	95.1	June 2012	Cross-sectional	To identify the strengths and weaknesses in NTP physicians’, nurses’ and nursing technicians’ comprehension of childhood TB in Lima, Peru
Colson 2010^25^	Harlem Hospital Chest Clinic, Manhattan, the USA	Candidate for LTBI treatment (majority non-US born)	251	100	2001 to 2004	Cross-sectional	To understand TB related knowledge, attitude and beliefs among candidates for LTBI treatment, especially the non-US born population.
Colson 2013^26^	30 TBESC clinics in the USA and Canada	Persons eligible for LTBI treatment	1762^a^	≈100	March 2007 to September 2008; October 2008 to May 2009	Cross-sectional	To identify modifiable factors associated with non-acceptance of LTBI treatment.
Coreil 2012^27^	TB Clinics under two county health departments in Florida, the USA	HCWs—doctors, nurses, social workers/case managers, nursing assistants, respiratory therapists, program administrators with medical backgrounds involved in delivery of TB services, health department TB service staff, hospital staff; Individuals diagnosed and treated for LTBI	133	100	Not reported	Cross-sectional	To compare TB-related stigma perceptions of health professionals with that of local patient populations and examine these in relation to other measures of anticipated distress
Cruz 2016^28^	Nationwide, the USA	HCWs—Pediatric infectious disease specialists	163	82.7	November 2015	Cross-sectional	To evaluate the extent to which advancements in the diagnosis and treatment of LTBI have been integrated into practice by paediatric infectious disease specialists
DeLuca 2018^29^	Byramjee Jeejeebhoy Government Medical College, Pune, India	Skin-test positive adult household contacts (HHC) of newly diagnosed adult pulmonary TB patients	100	100	December 2015 to March 2017	Cross-sectional	To survey the perceptions of TB infection and prevention among skin-test positive individuals
Evenblij^b^ 2016^30^	Nationwide, Netherlands	HCWs—HIV physicians	Questionnaire 1:51 Questionnaire 2:24	100 100	January to May 2014	Mixed-method	To assess intention of HIV physicians to screen for LTBI and offer preventive therapy
Gao 2015^b^^31^	British Columbia Centre for Disease Control TB clinics, Canada	Foreign-born individuals (Chinese immigrants) treated for LTBI	912	100	June 2013 to June 2014	Mixed-method	To document knowledge levels of patients in a TB clinic; to identify Chinese immigrants’ knowledge and perceptions of LTBI
Gupta 2011^32^	Nationwide, Australia	HCWs—Gastroenterologist	44	100	Not reported	Cross-sectional	To survey practice among gastroenterologist in Australia relating to screening for LTBI and vaccination of patients with inflammatory bowel disease prior to treatment with TNF-α inhibitors.
Gutsfeld 2014^10^	Nationwide, Germany	HCWs—Pulmonologists, public health officers, rheumatologists, physicians in HIV and occupational medicine	510^a^	≈100	Not reported	Cross-sectional	To evaluate knowledge and attitude of physician decision makers in Germany about current methods for the diagnosis of LTBI and preventive chemotherapy
Hill 2010^33^	Seven high schools in San Diego County, California, the US	Adolescents treated for LTBI Parents to adolescents who were treated for LTBI	570^a^	≈100	Not reported	Cross-sectional	To describe the barriers to effective and timely LTBI treatment encountered in a research study on isoniazid adherence in adolescents
Hirsch-Moverman 2006^34^	Two teaching hospitals in New York, the USA	HCWs—Medical house staff receiving training	162^a^	≈98.2	2001–2002	Cross-sectional	To compare beliefs and intentions about LTBI treatment among IMG and US medical graduate (USMG) physicians-in-training
Hirsch-Moverman 2013^35^	Denver, Colorado; Houston, Texas; Baltimore, Maryland and New York City, New York, the USA	HCWs—Nursing, administrative/clerical, medical staff, research scientist, lab technician, therapist	406	100	Not reported	Cross-sectional	To determine and compare the acceptability of the TST and IGRAs for use in HCWs undergoing routine LTBI testing.
Howley 2015^36^	Health departments in the city of Dallas, Houston, Philadelphia, New York and Washington DC; the states of Maryland, New Jersey, North Carolina and Virginia, and urban jurisdictions within the states of Georgia and Tennessee, the USA	TB patients	477	100	16 December 2009 to 31 March 2011	Cross-sectional	To describe TB KAB of U.S.-born TB patients and to explore TB knowledge, attitude and behaviour differences between black and white racial groups
Jackson 2007^37^	20 National TB Curriculum Consortium (NTCC) Schools, the USA	Health care students	1480^a^	≈100	2005	Cross-sectional	To assess students for basic knowledge of TB and how confident they feel about taking care of persons with LTBI or active TB
Kane 2013^38^	Two TB outpatient conics in St James Hospital, Dublin, Ireland	Individuals treated for LTBI, including foreign-born communities (from TB endemic country), hospital worker, contact of active TB case, pre-biological agents	143	98.6	Not reported	Cross-sectional	To identify patients’ views of LTBI and potential barriers to acceptance and completion of treatment
Karakousis 2007^39^	Baltimore and Philadelphia, the USA	HCWs—Medical residents training at the US urban medical centres	131	100	Final month of academic year, 2005	Cross-sectional	To assess medical resident knowledge about TB diagnosis and early management based on American Thoracic Society guidelines
Lazar 2010^40^	Connecticut, the USA	HCWs—Physicians	258	74.8	February to April 2008	Cross-sectional	To identify current physician practices related to testing school children for TB in the state of Connecticut.
Li 2018^41^	Shanghai, China	TB contacts (student populations)	560^a^	≈100	May 2016 to Feb 2017	Cross-sectional	To assess level of knowledge of student contacts about TB, their willingness to participate in LTBI chemo-prophylaxis if diagnosed with LTBI after the contact and concerns associated with the acceptance of LTBI treatment
Mirtskhulava 2015^42^	Nationwide, Georgia	HCWs–Physicians and nurses from Georgian NTP involved in TB programs; with experience in TB	240	100	July to December 2011	Cross-sectional	To understand TB infection control in healthcare facilities in Georgia
Montagna 2014^44^	15 universities in urban areas across Italy	Health care students in nursing and medicine courses	2200	100	October 2012 to June 2013	Cross-sectional	To determine the level of knowledge of TB and its control measures among undergraduate health care students in Italy and to investigate personal experiences with practices to prevent TB infection
Montagna 2018^43^	17 universities across Italy	Medical and health professional students—Medicine, dentistry, nursing, health assistants, physiotherapists, obstetricians	5209	100	March to April 2018	Cross-sectional	To determine the level of knowledge about Mantoux TST among health care students in Italy
Moolphate 2013^45^	Upper northern region of Thailand	HCWs—Physicians, TB clinic nurses, HIV clinic nurses; with experience in TB	198^a^	≈72.7 to 100	July to September 2012	Cross-sectional	To assess the barriers and motivations for the implementation of IPT for PLWH in upper northern Thailand
Narayanan 2019^46^	New Jersey, the USA	Non-US born South Asian community members (from high TB burden country/TB endemic country)	446 (387 included in analysis)	96.3	March to June 2018	Cross-sectional community survey	To assess the knowledge, attitudes and health behaviours of the New Jersey non-US-born South Asian community relating to testing and treatment of LTBI
O’Donnell 2011^47^	Boston University Medical Centre, the USA	Patients with HCV and HIV co-infection	230	100	January 2007 to March 2008	Cross-sectional	We hypothesized that a population of TB high-risk urban subjects, the majority of injection drug users, would prefer QFT-G testing over TST and would be more likely to accept LTBI diagnosis and treatment based on QFT-G results.
Pathak 2016^48^	Liverpool Hospital (with a major TB clinic), Sydney, Australia	HCW—Doctors and nurses from respiratory, medical or surgical departments	311^a^	≈97.8	Not reported	Cross-sectional	To examine doctors' and nurses' experience of TB screening and to explore their attitudes towards preventive TB treatment
Quirós 2018^49^	Multicentre study in Spain	HCWs—Doctors from Dermatology, Rheumatology, Digestive Diseases, Internal Medicine, Infectious Diseases, and Pulmonology	747	100	Not reported	Cross-sectional	To determine specialists’ adherence to the consensus documents on the prevention and treatment of TB in patients who are candidates for biological treatment in Spain
Ramos 2018^50^	Primary health clinics in Recife, Manaus, Rio de Janeiro, Brazil	HCWs—Physicians and nurses; with formal training in TB	101	100	January 2015 to July 2016	Cross-sectional	To explore the perspectives of primary care physicians and nurses regarding TB transmission and prevention, using knowledge, attitude and practices survey
Salazar-Schicchi 2004^51^	Harlem Hospital Centre, Manhattan, New York, the US	HCWs—Physicians in training, all international medical graduates from TB endemic countries	77^a^	≈100	January 2001	Cross-sectional	To investigate the attitudes of international medical graduates (IMGs) about treatment of LTBI
Skinner 2013^52^	Western Cape, South Africa	Care givers of children aged 0–5 years referred for IPT, following household contact with adult TB case	36	100	June to October 2012	Cross-sectional survey	Describe knowledge, attitude and intended behaviours in care givers of young children referred for IPT in a resources-constrained setting with high TB rates
Smith 2012^53^	24 European Union countries	HCWs—Prescribers (rheumatologists, gastroenterologists and dermatologists) of anti-TNF agents	915	100	1 March to 31 May 2010	Cross-sectional	To assess the awareness of TB risk, performance of TB screening and factors predicting TB screening among prescribers of TNF-α agents
Spruijt 2020^b^^54^	Five Public Health Services in Netherlands	HCWs—TB physicians, TB nurses; with experience in TB Foreign-born individuals (immigrants not applying for asylum) who were eligible for LTBI screening	94	100	March to September 2016	Mixed method	To evaluate barriers to LTBI treatment: language barriers, reasons for not initiating, discontinuing or not completing LTBI treatment, the occurrence of side effects, other challenges encountered during the treatment and the frequency of supervision
Stout 2006^55^	North Carolina, the US	HCWs—Paediatricians and family practitioners	149	100	Not reported	Cross-sectional	To understand physicians’ knowledge and attitudes toward the treatment of young children with LTBI in a low-incidence region
Trajman 2019^56^	18 clinics in Rio de Janeiro, Manaus and Recife Brazil	HCWs—Auxiliary primary healthcare workers (community health agents and nurse aids)	135	100	May 2015 to January 2016	Cross-sectional	To understand auxiliary primary healthcare workers’ knowledge, attitudes and practices (KAP) regarding contact investigation in Brazil
Tran 2017^57^	Nationwide, the US	HCWs—Rheumatologists	768^a^	100	Not reported	Cross-sectional	To understand the current practice patterns of both the USA and international members of the American College of Rheumatology (rheumatologists) in screening and managing LTBI in patients treated with biologics
Vinnard 2012^58^	American College for Occupational and Environmental Medicine, the US	HCWs—Physician members of the Medical Centre Occupational Health	103	88.8	Not reported	Cross-sectional	To determine current practices for diagnosing LTBI by occupational physicians in healthcare settings, and to understand attitudes on the selection of IGRAs versus TSTs
Walker 2018^59^	Two sites for English for Speakers of Other Languages (ESOL) courses, England, the UK	Students from countries with high TB incidence	154^a^	≈89.5	Not reported	Cross-sectional	To describe an outreach programme targeted at recent immigrants enrolled in ESOL programmes at a Birmingham community college (CC) using participatory and asset-based community development approaches
Xerinda 2016^60^	Nationwide, Portugal	HCWs—Physicians (specialist and trainees), including rheumatologists, dermatologists and gastroenterologists	95	100	Not reported	Cross-sectional	To identify perceived barriers to TB screening among patients to anti-TNF treatment
Yates 2015^61^	Hahnemann University Hospital and Cooper University Hospital, the US	HCWs—Resident physicians	138^a^	≈98.6	Not reported	Cross-sectional	To measure differences in LTBI treatment attitudes among resident physicians when diagnosis is established with a positive TST, as compared with a positive IGRA
Zhou 2014^62^	Zhengzhou Central Hospital (non-TB) and Henan Provincial Infectious Disease Hospital (TB)	HCWs—Not specified	731 (94 included in analysis)	100	January to December 2011	Cross-sectional	To determine the impact factors of LTBI and the knowledge of TB prevention and treatment policy among HCWs in different types of hospitals

TB: tuberculosis; the US: The United States; LTBI: latent tuberculosis infection; HCW: healthcare worker; the UK: The United Kingdom; TNF: tumour necrosis factor; TBESC: Tuberculosis Epidemiologic Studies Consortium; HIV: human immunodeficiency virus; TST: tuberculin skin test; IGRA: interferon gamma release assay; NTP: National Tuberculosis Programme; IPT: isoniazid preventive therapy; PLWH: people living with HIV; HCV: hepatitis C virus; QFT-G: QuantiFERON-TB Gold

^a^Some questions did not receive full response, but the questions were not specified

^b^In the mixed method study, only the survey-based section of the study was extracted for this review.

### Risk of bias reporting

Most of the studies were rated to have a low risk of bias (*n* = 35)^10^^,^^17–27^^,^^29^^,^^31^^,^^33–35^^,^^38^^,^^39^^,^^41–44^^,^^46^^,^^47^^,^^49–54^^,^^56^^,^^60–62^, and 12 studies had a moderate level of risk of bias^28^^,^^30^^,^^32^^,^^36^^,^^37^^,^^40^^,^^45^^,^^48^^,^^55^^,^^57–59^ (Supplementary Appendix 7). The risk of bias primarily arose from a lack of reporting on the sampling frame (*n* = 28)^10^^,^^17^^,^^28–30^^,^^32–34^^,^^37^^,^^38^^,^^40^^,^^41^^,^^43–48^^,^^50^^,^^52–55^^,^^57–59^^,^^61^^,^^62^ and the sampling method where convenience sampling or voluntary sample was used (*n* = 20).^17^^,^^21^^,^^22^^,^^27^^,^^29–31^^,^^33^^,^^36^^,^^38^^,^^42–44^^,^^46^^,^^47^^,^^49^^,^^50^^,^^52^^,^^56^^,^^58^ Hence, there was uncertainty on whether the true close representation of the target population was established. Moreover, half of the studies (*n* = 23) did not use pilot-tested and validated study instruments for data collection.^20^^,^^21^^,^^23^^,^^25^^,^^26^^,^^30^^,^^32–38^^,^^41^^,^^47–49^^,^^52^^,^^55^^,^^57^^,^^61^^,^^62^ This could potentially affect the reliability, consistency and accuracy of data collected.

### Barriers to LTBI management

**Fig. 2:**
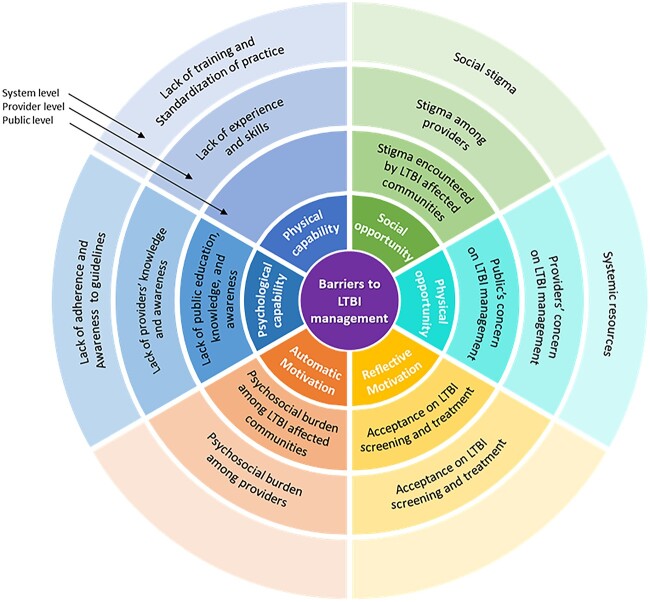
Barriers to LTBI management at system, provider and general public level mapped on to the subcomponents of COM-B model.

To aid in the interpretation and implementation of our results, we categorized the barriers to LTBI management into the public, provider or system level (Fig. 2).^4^ The specific details of each domain and number of studies were summarized in Table 2 and Supplementary Appendix 8. A narrative summary is presented below.

**Table 2:** Overview of results: Summary of barriers with corresponding intervention functions across levels (public, provider, and system) and theoretical component

COM-B subcomponent	General public level			Provider level			System level		
	Barriers	Studies	Intervention function	Barriers	Studies	Intervention function	Barriers	Studies	Intervention function
Physical capability (PhC)	Not reported			Lack of experience in LTBI screening	[10, 19, 28, 43, 44]	Training	Lack of standardization in LTBI practice (screening, treatment) for patients requiring anti-TNF therapies	[23, 32, 49, 53, 57, 60]	Training
				Lack of experience in LTBI treatment	[19, 49]	Environment restructuring	Lack of standardization and guidance in LTBI practice (screening, treatment) for children population	[28, 40, 55]	Environment restructuring
				Lack of experience in LTBI management (in general)	[28, 33, 37, 55]	Modelling	Lack of standardization in LTBI practice (screening, treatment) in general	[10, 33, 58]	Modelling
							Lack of training and/or training availability	[24, 45, 50, 55, 56, 60]	
Psychological capability (PsC)	Knowledge about LTBI characteristic: LTBI cannot be spread	[21, 22, 25, 26, 29, 31, 36]	Education	Knowledge about LTBI characteristic: LTBI cannot be spread	[18]	Education	Lack of adherence to guidelines on LTBI screening	[23, 30, 40]	Training
	Knowledge about LTBI characteristic: LTBI is asymptomatic	[22, 31, 36]	Environment restructuring	Knowledge about LTBI characteristic: LTBI is asymptomatic	[24]	Training	Lack of adherence to guidelines on LTBI treatment	[23, 40, 45, 53]	Environment restructuring
	Knowledge about LTBI characteristic: LTBI can detected using skin test (TST)/ blood test (IGRA); TST positive could be an indication of LTBI and not confirmation for TB disease	[21, 25, 29, 33, 36]		Knowledge about LTBI characteristic: LTBI can detected using skin test (TST)/ blood test (IGRA); TST positive could be an indication of LTBI and not confirmation for TB disease	[18, 24, 39, 44, 50, 56]	Environment restructuring	Lack of awareness of LTBI guidelines and/or policies	[39, 40, 43, 45, 60]	Modelling
	Knowledge about LTBI: To differentiate between TB and LTBI	[22, 29]		Knowledge about LTBI: To differentiate between TB and LTBI	[48, 54, 56]		Unclear direction of national policy for IPT program	[45]	
	Knowledge about LTBI in general	[17, 33]		Knowledge about LTBI in general	[39, 42]				
	Misperception about BCG: BCG vaccine can prevent or protect against TB/ LTBI; with BCG vaccine, a person cannot develop TB/ LTBI.	[17, 21, 25]		Misperception about BCG: BCG vaccine can prevent or protect against TB/ LTBI; with BCG vaccine, a person cannot develop TB/ LTBI.	[18, 44, 51]				
	Misperception about BCG: With BCG vaccine, LTBI screening/ treatment is not needed	[26, 59]		Misperception about BCG: With BCG vaccine, LTBI screening/ treatment is not needed	[20, 33, 34, 39, 48]				
	Misperception about BCG: BCG vaccine caused positive TST	[33]		Misperception about BCG: BCG vaccine caused positive TST, hence LTBI treatment is not required	[33, 34, 39, 51]				
	Misperception about BCG: BCG vaccine provides lifelong immunity	[31, 46]		Misperception about BCG: TST is contraindicated to BCG vaccine	[37, 55]				
	Awareness about the purpose of LTBI screening	[59]		Knowledge about types of screening test	[18, 23, 35]				
	Knowledge about information on LTBI treatment in general	[17, 29, 31, 36, 52]		Lack of confidence in LTBI screening	[19]				
	Knowledge about LTBI treatment rationale	[22, 31, 33, 36, 41, 52]		Knowledge about information on LTBI treatment in general	[10, 24, 33, 39, 49, 50]				
	Understanding on the risk of TB activation among specific high-risk population	[36, 38, 41, 52]		Knowledge about LTBI treatment rationale	[34, 38, 42, 45, 50, 51, 56]				
				Lack of confidence in LTBI treatment	[19]				
				Risk of progression from LTBI to TB disease	[24, 37, 39, 42, 49, 50, 53]				
				Knowledge about LTBI management in children	[24, 50, 55]				
Reflective motivation (RM)	Participants do not think they had TB germ, as they do not feel sick	[25, 26, 59]	Education	Hesitancy to provide LTBI screening in general	[10, 30, 32]	Education	The need of LTBI testing and treatment policy	[19, 30, 44, 49]	Environment restructuring
	Participants do not think they had the risk of LTBI	[26, 41]	Environment restructuring	Hesitancy to provide screening to specific high-risk populations	[30, 42, 50, 56]	Environment restructuring			Incentivization
	Participants do not think they had higher risk of progressing to active TB from LTBI	[29]	Persuasion	Testing preference: IGRA (accuracy of test results)	[30, 35, 61]	Persuasion			Persuasion
	Testing preference: IGRA	[47]	Incentivization	Hesitancy to LTBI screening for participants themselves	[42, 48]	Incentivization			Modelling
	Hesitancy to LTBI screening	[46]	Modelling	Hesitancy to provide LTBI treatment to high-risk population	[19, 34, 48, 51, 54]	Modelling			
	Hesitancy to initiate LTBI treatment	[29, 30, 41]	Enablement	Hesitancy to accept and initiate LTBI treatment for participants themselves upon LTBI diagnosis	[20, 42, 48, 58, 61]	Enablement			
	Hesitancy to complete LTBI treatment	[38, 52]		Perception of the risk of LTBI treatment is higher than its benefits	[34, 42, 51, 54]				
	Perception on negative health consequences with LTBI treatment	[36]		Perception of the risk of getting resistant strain with LTBI treatment	[42, 45]				
	Lack of trust in health care system	[26]		Perception on the unobservable effects of medication, hence LTBI treatment is unnecessary	[42, 48, 54]				
	Participants dislike taking medicines	[26]		Perception on the ability to self-monitor, where LTBI treatment is not needed	[48]				
Automatic motivation (AM)	Embarrassment/ feeling ashamed about having LTBI	[21, 25, 36]	Education	Embarrassment/ feeling ashamed about having LTBI	[27]	Education	Not reported		
			Modelling			Modelling			
	Sadness, worry, stress, anxious	[27, 33, 36, 41]	Enablement	Sadness, worry, stress, anxious	[27, 42, 62]	Enablement			
Social opportunity (SO)	Stigma	[27, 52, 56]	Education	Stigma	[27]	Education	Social stigma	[27]	Enablement
			Enablement			Enablement			
	Concerns about what friends and family think about LTBI, and its treatment	[21, 25, 36]		Anxiety regarding family	[27]				
	Testing migrants is discriminative	[59]							
Physical opportunity (PO)	Concerns on side effects of LTBI treatment	[10, 25, 26, 33, 38, 41, 47, 52]	Education Environment restructuring Incentivization	Concerns on side effects of LTBI treatment	[10, 20, 45, 48, 54]	Education Environment restructuring Incentivization	Lack of availability of primary care-based GP-led service for LTBI treatment for adult migrants	[19]	Training Environment restructuring Incentivization
	Duration of LTBI treatment	[26, 38, 41]		Duration of LTBI treatment	[10, 20, 54]		Lack of timely access to specialist TB services and support by GPs.	[19]	Persuasion
	Work responsibility; time constraint; amount of time spent for medical services	[25, 33, 38]		Poor adherence to LTBI treatment	[45]		Lack of resources such as availability of appointments and healthcare providers from multidisciplinary background in LTBI management	[19]	
	Cost of medical service and charges	[33, 41]		Concerns on side effects from LTBI testing tools	[35, 62]		Healthcare providers were too busy with work responsibility than to manage LTBI	[56]	
	Logistic difficulty: Transport issue	[33]		Concerns on the cost of LTBI screening	[35]		Potentially a lack of funding	[19, 28, 33]	
	Cost of travel to receive medical service	[38]		Concerns on the pain from LTBI screening	[35]		Lack of incentives for healthcare providers who adhere to guidelines	[30]	
	Pills hard to swallow	[25]		Concerns on previous positive results, hence not to be screened again	[62]		Cost and efficacy of diagnostics	[10, 58]	
	Lack of self-efficacy: Follow doctor’s advice if to initiate LTBI treatment or otherwise	[26, 41]		Unable to attend medical appointment for LTBI	[54]		Availability of LTBI screening tests: TST and/or IGRA	[23, 28, 58]	
				Work responsibility, hence unable to attend LTBI training	[24]		Treatment efficacy	[10]	

LTBI: latent tuberculosis infection; TNF: tumour necrosis factor; TST: tuberculin skin test; IGRA: interferon gamma release assay; TB: tuberculosis: IPT: isoniazid preventive therapy: BCG: Bacillus Calmette–Guérin

### Physical capability

One of the most commonly identified barriers to LTBI management was the lack of physical capability among healthcare providers, where they did not have sufficient exposure and experience in handling LTBI.^10^^,^^19^^,^^28^^,^^33^^,^^37^^,^^43^^,^^44^^,^^49^^,^^55^ This could have explained the lack of standardization in LTBI practice.^28^^,^^33^^,^^49^ The need of LTBI training and/or training availability for healthcare providers was identified as an opportunity to enhance their skills in managing LTBI cases.^24^^,^^45^^,^^50^^,^^55^^,^^56^^,^^60^

### Psychological capability

A lack of understanding of LTBI was identified as another opportunity for improvement among the public and healthcare providers. The review found that many had poor understanding of LTBI clinical characteristics,^10^^,^^17–19^^,^^21–26^^,^^29^^,^^31^^,^^33–36^^,^^38^^,^^39^^,^^41^^,^^42^^,^^44^^,^^45^^,^^48–52^^,^^54^^,^^56^^,^^59^ role of Bacillus Calmette–Guérin (BCG) vaccination^17^^,^^18^^,^^20^^,^^21^^,^^25^^,^^26^^,^^31^^,^^33^^,^^34^^,^^37^^,^^39^^,^^44^^,^^46^^,^^48^^,^^51^^,^^55^^,^^59^ and the risk factors for TB reactivation.^24^^,^^36–39^^,^^41^^,^^42^^,^^49^^,^^50^^,^^52^^,^^53^ For example, one common misconception was that BCG vaccination was often thought to confer lifelong immunity, even among healthcare workers.^17^^,^^18^^,^^21^^,^^25^^,^^31^^,^^36^^,^^44^^,^^51^ This could have led to poor compliance and adherence to LTBI treatment and management guidelines.

### Reflective motivation

Reflective motivation was another aspect which could be targeted in improving LTBI screening, diagnosis and treatment. Screening and testing hesitancy for LTBI was noted especially among the general public as they were not aware of the risk of LTBI and TB reactivation.^10^^,^^25^^,^^26^^,^^29^^,^^30^^,^^32^^,^^41^^,^^42^^,^^46^^,^^48^^,^^50^^,^^56^^,^^59^ Similarly, LTBI treatment was not well accepted by the general public and healthcare providers, as they were concerned about the effectiveness and risk of LTBI treatment.^19^^,^^20^^,^^26^^,^^29^^,^^30^^,^^34^^,^^36^^,^^38^^,^^41^^,^^42^^,^^45^^,^^48^^,^^51^^,^^52^^,^^54^^,^^58^^,^^61^ As such, there is a need to enforce LTBI testing and treatment policy to guide appropriate decision-making in LTBI practice.^19^^,^^20^^,^^44^^,^^49^

### Automatic motivation

LTBI was often associated with psychosocial burden, such as worries, stress, anxiety and embarrassment.^21^^,^^25^^,^^27^^,^^33^^,^^36^^,^^41^^,^^42^^,^^62^ It was reported that healthcare workers who had LTBI suffered from higher anticipated psychosocial distress compared to public who had LTBI.^27^ To address this, better support system could be established to ensure the psychological, mental, emotional and social well-being of individuals with LTBI.

### Social opportunity

Stigma with the fear of judgement has greatly impacted the health and well-being of LTBI affected communities.^21^^,^^25^^,^^27^^,^^36^^,^^52^^,^^56^ The concerns arose during their treatment process and clinic visits, with the worries of being discriminated by their community. This social impact was more evident among healthcare providers and migrant communities because of their social status.^27^^,^^59^

### Physical opportunity

The low acceptability of LTBI treatment was primarily attributed to the concerns over the side effects and prolonged treatment duration for this asymptomatic condition.^10^^,^^20^^,^^25^^,^^26^^,^^33^^,^^38^^,^^41^^,^^45^^,^^47^^,^^48^^,^^52^^,^^54^ In addition, the lack of resources and support for healthcare providers in LTBI management such as funding and screening availability has to be overcome in ensuring the continuity and good quality of LTBI services.^19^^,^^23^^,^^28^^,^^33^^,^^58^

### Using behaviour change wheel to address LTBI barriers

In response to the barriers above, seven of the nine intervention functions in the BCW, namely education, persuasion, incentivization, training, environmental restructuring, modelling and enablement, were selected to guide improvement in LTBI management (Table 2 & Supplementary Appendix 9).^4^

### Physical capability

As healthcare providers reported a lack of experience and standardization in LTBI practice, additional training should be conducted. Some topics of interest should include identification of LTBI, especially for the high-risk populations.^23^^,^^28^^,^^32^^,^^40^^,^^49^^,^^53^^,^^55^^,^^57^^,^^60^ From the perspective of environment restructuring, prompts and cues such as an LTBI checklist can be prepared for healthcare providers attending individuals at risk of LTBI.^23^^,^^28^^,^^32^^,^^40^^,^^49^^,^^53^^,^^55^^,^^57^^,^^60^ In addition, faculty mentoring or modelling can be implemented. Experienced senior healthcare providers could be the mentors to juniors in managing LTBI cases.

### Psychological capability

Many individuals have a misunderstanding towards LTBI.^10^^,^^17–19^^,^^21–26^^,^^29^^,^^31^^,^^33–39^^,^^41^^,^^42^^,^^44^^,^^45^^,^^48–56^^,^^59^***Education*** was identified to be an important intervention to improve healthcare providers and the general public’s awareness towards LTBI. While they are better informed with the importance of LTBI screening and treatment, this helps to encourage their acceptance of LTBI care. ***Education*** can be delivered in the forms of community campaigns, regular seminar, courses and professional ***training***. This can go hand in hand with an ***environment restructuring,*** such as public health campaigns through mass media. Other opportunities include the use of influencers and advocators who could act as the role model ***(modelling)***, to raise public awareness towards LTBI.

### Reflective motivation

One key strategy towards the eradication of TB and LTBI is the need for LTBI screening and treatment uptake. ***Education***, ***environment restructuring*** and ***persuasion*** can be applied to encourage eligible individuals to accept LTBI screening and treatment.^10^^,^^19^^,^^20^^,^^25^^,^^26^^,^^29^^,^^30^^,^^32^^,^^34^^,^^36^^,^^38^^,^^41^^,^^42^^,^^45^^,^^46^^,^^48^^,^^50–52^^,^^54^^,^^56^^,^^58^^,^^59^^,^^61^ Relevant information on LTBI could be shared through verbal discussion together with visual aids such as infographics or pamphlets. Given that many will experience catastrophic costs, ***incentivization*** such as food or travel vouchers can be provided to individuals accepting LTBI screening and treatment.^38^ Incentives can also be offered to healthcare providers who attend to LTBI cases.^10^^,^^19^^,^^20^^,^^30^^,^^32^^,^^34^^,^^42^^,^^45^^,^^48^^,^^50^^,^^51^^,^^54^^,^^56^^,^^58^^,^^61^ Meanwhile, ***enablement*** allows psychosocial support services, counselling sessions or social support network to be given to individuals with LTBI. This is where ***modelling*** can be applied as well, to which LTBI treatment completers can be the mentors to support those who are new to the treatment.^20^^,^^29^^,^^30^^,^^36^^,^^38^^,^^41^^,^^42^^,^^48^^,^^52^^,^^58^^,^^61^

### Automatic motivation

Individuals with LTBI including healthcare providers were troubled by psychosocial impact due to LTBI.^21^^,^^25^^,^^27^^,^^33^^,^^36^^,^^41^^,^^42^^,^^62^ This could be attributed to the misunderstanding and misperception towards LTBI. With community ***education*** on LTBI, general public and healthcare providers can be better informed about LTBI. This helps to overcome the stress, anxiety and embarrassment associated with LTBI. This intervention can be coupled with ***enablement*** for psychosocial support to be provided to individuals with LTBI. In addition, ***modelling*** helps to alleviate distress among individuals with LTBI, where individuals who have undergone LTBI screening and treatment could be the guidance for those who are new to LTBI care.

### Social opportunity

As stigma is one of the concerns for healthcare providers and individuals with LTBI,^21^^,^^25^^,^^27^^,^^36^^,^^52^^,^^56^^,^^59^***education*** and ***enablement*** are essential strategies to cope with potential discrimination stem from LTBI. ***Education*** can be conducted through community campaigns for better public awareness, whereas ***enablement*** in the form of social support can be offered not only to individual with LTBI but also their caregivers, family and close friends. Knowledge coupled with social support can work synergistically in tackling stigma in LTBI.

### Physical opportunity

In response to the concerns over LTBI treatment,^10^^,^^20^^,^^24–26^^,^^33^^,^^35^^,^^38^^,^^41^^,^^45^^,^^47^^,^^48^^,^^52^^,^^54^^,^^62^ targeted ***education*** can be reinforced through ***training*** for the healthcare providers. They could in turn share the information ***(education)*** with individuals eligible for LTBI treatment during the consultation and counselling sessions. To address the issues on time constraint, financial and logistic challenges in accessing LTBI care, ***incentivization*** in the forms of food and travel vouchers or financial aids can be provided to subsidize the direct non-medical costs needed to access LTBI services.^33^^,^^38^ Remuneration can be offered to healthcare providers and healthcare facilities that provide LTBI care with strict adherence to LTBI guidelines.^30^ In addition, ***persuasion*** can be applied to encourage healthcare providers to actively participate in LTBI practice and training. ***Environment restructuring*** will also be needed to ensure sufficient funding is available to increase the coverage of LTBI screening in local health facilities.^19^^,^^23^^,^^28^^,^^33^^,^^58^

## Discussion

### Main finding of this study

To our best knowledge, this is the first systematic review incorporating theoretical rationale with the COM-B model to identify the gaps in LTBI management. The complex structure of barriers to LTBI management can be summarized as suboptimal knowledge (physical capability) and misperception of LTBI (reflective motivation and physical opportunity), stigma (social opportunity) and psychosocial burden (automatic motivation). As the barriers of LTBI management were inter-related with one another, our findings suggest the need of a multifaceted approach guided by the intervention functions from the BCW to address the shortfall in LTBI care.

### What is already known on this topic

Active TB disease continues to persist in many countries, largely because of the on-going reactivation of LTBI. As such, TB elimination will require extensive screening and treatment of LTBI. The primary benefit of LTBI treatment is that it can effectively reduce the risk of developing active TB disease in specific high-risk populations. Unfortunately, this is not often seen among the individual(s) with LTBI and their communities. The gaps in LTBI management were known to be attributed to individual, provider and systemic factors.^7^^,^^8^ These include patients’ worries about the negative impact of LTBI treatment, a lack of knowledge among healthcare providers in LTBI testing and treatment and a low prioritization of LTBI management in the healthcare system.^7^ In view of these, a holistic view of the barriers to LTBI management would be beneficial for improvements targeting individual (patient and public), provider and system levels to be formulated.

### What this study adds

We found that holistic strategies targeting knowledge, socio-economic and cultural gaps should be adopted to facilitate the implementation of LTBI management. In order to achieve this, the BCW was used as an inclusive and holistic guidance in pivoting interventions for behaviour change at various levels, including the policies, service, healthcare providers, patients and the public. The BCW also incorporates socio-economic and cultural aspects into its intervention functions to address the social determinants of health that play an important role in health outcomes.^4^ For example, low acceptability to LTBI treatment could be attributed to the misunderstanding on the protective effect of BCG vaccine (psychological capability),^17^^,^^18^^,^^20^^,^^21^^,^^25^^,^^26^^,^^31^^,^^33^^,^^34^^,^^37^^,^^39^^,^^44^^,^^46^^,^^48^^,^^51^^,^^55^^,^^59^ misperception on the risk of treatment (reflective motivation),^19^^,^^20^^,^^26^^,^^29^^,^^30^^,^^34^^,^^36^^,^^38^^,^^41^^,^^42^^,^^45^^,^^48^^,^^51^^,^^52^^,^^54^^,^^58^^,^^61^ concerns over the side effects of medications (physical opportunity),^10^^,^^20^^,^^25^^,^^26^^,^^33^^,^^38^^,^^41^^,^^47^^,^^52^^,^^54^ psychosocial distress (automatic motivation)^21^^,^^25^^,^^27^^,^^33^^,^^36^^,^^41^^,^^42^^,^^62^ and the fear of stigma (social opportunity).^21^^,^^25^^,^^27^^,^^36^^,^^52^^,^^56^^,^^59^ Using BCW, ***education***, ***environment restructuring***, ***persuasion*** and ***modelling*** were identified to address the misunderstanding, misperception and concerns over LTBI treatment. ***Incentivization*** and ***enablement** were* equally important to provide financial and social support to overcome the distress and stigma among individuals with LTBI. Hence, a combination of intervention functions was required to resolve the underlying barriers to LTBI treatment hesitancy.

While there is no standalone intervention in addressing the barriers, it was observed that education is the fundamental strategy to target behaviour change in LTBI management. Nevertheless, we need to take the study setting into consideration. For example, there are differences in resource distribution and healthcare priorities in high TB burden countries compared to low-incidence countries.^63^^,^^64^ Therefore, there is a need to tailor appropriate educational modules for different settings, in order to optimize the impact of educational intervention.

As the BCW targets changes and improvements across various levels, this implies the importance of multidisciplinary collaboration to tackle the barriers in LTBI management. However, the role of community personnel such as community healthcare workers and pharmacists in LTBI management has not been fully explored. We have only identified one study which recruited community healthcare workers,^56^ and one study that recruited pharmacists as part of the study cohort.^18^ The participation of healthcare providers from all disciplines for LTBI ***training*** should be encouraged to share the professional responsibilities in providing quality LTBI care across all levels of health service delivery.

This review has highlighted the importance of collective efforts from all parties to improve LTBI care. Overall, it is essential to raise the awareness of active TB disease and LTBI as a public health emergency and increase political commitment to this neglected disease. This is to ensure that TB prevention and control can be achieved, in accomplishing the targets of ending global TB epidemic by the next decade.

### Limitations of this study

There are several limitations which need to be considered. First, we included only articles published in English. As such, relevant publications in other languages might have been missed out, despite our extensive literature search. Furthermore, the substantial heterogeneity in the included studies, in terms of research aims, sampling techniques, questionnaire designs, questionnaire validation process and response rates could potentially affect the quality of overall data. Non-response bias, attrition bias and the possibility of self-selecting bias among the participants might be inevitable in survey studies. We conducted the risk of bias assessment meticulously, where none of the studies was reported to have a high risk of bias.

Since most of the studies were conducted in low-incidence countries,^10^^,^^17^^,^^19–23^^,^^25–28^^,^^30–40^^,^^43^^,^^44^^,^^46–48^^,^^51^^,^^54^^,^^55^^,^^57–59^^,^^61^ the results may not be generalizable especially in countries with high TB burden as they may have different priorities in TB control due to constraints of resources. Indeed, the small number of studies in countries with high TB burden may reflect the suboptimal advocacy in LTBI care.^24^^,^^29^^,^^41^^,^^45^^,^^50^^,^^52^^,^^56^^,^^62^

## Conclusion

The findings from this review highlight potential strategies to better guide changes and improvements in policies for better delivery of LTBI management. Through a successful implementation of LTBI care, this can serve as an important stepping stone to accomplish the milestone of WHO END TB Strategy.

## Conflict of interest

The authors declare no conflict of interest.

## Data availability

All data are incorporated into the article and its online supplementary material.

## Supplementary Material

Appendix_R1_fdad051

## Notes

**Yen Jun Wong**, PhD, Assistant Lecturer

**Khuen Yen Ng**, PhD, Senior Lecturer

**Shaun Wen Huey Lee**, PhD, Professor
