# Supplementary material for: How can we improve latent tuberculosis infection management using behaviour change wheel: a systematic review
Source: J Public Health (Oxf). 2023 May 5;45(3):e447–66. doi: 10.1093/pubmed/fdad051 (PMC10470485; doi:10.1093/pubmed/fdad051)
Supplement: Appendix_R1_fdad051 [file appendix_r1_fdad051.docx]

**Appendix**

**How Can We Improve Latent Tuberculosis Infection Care Using Behaviour Change Wheel: A Systematic Review**

Yen Jun Wong, Khuen Yen Ng, Shaun Wen Huey Lee

Contents

[Appendix 1 Preferred Reporting Items for Systematic Reviews and Meta-Analyses (PRISMA) checklist 2](#_Toc124149056)

[Appendix 2 Eligibility criteria 5](#_Toc124149057)

[Appendix 3 Search strategy 6](#_Toc124149058)

[Appendix 4 List of excluded studies 14](#_Toc124149059)

[Appendix 5 Risk of bias assessment checklist 15](#_Toc124149060)

[Appendix 6 Summary of study characteristics 18](#_Toc124149061)

[Appendix 7 Risk of bias of the included studies 20](#_Toc124149062)

[Appendix 8 Findings of included studies by KAP category, level (general public, provider, system), and sub-component (COM-B) 22](#_Toc124149063)

[Appendix 9 Figure S1 Intervention to improve latent tuberculosis management at system, provider and general public level mapped on to the subcomponents of Capability, Opportunity, and Motivation-Behaviour (COM-B) model 63](#_Toc124149064)

[References 64](#_Toc124149065)

# Appendix 1 Preferred Reporting Items for Systematic Reviews and Meta-Analyses (PRISMA) checklist

Table S1.1 PRISMA checklist

| **Section and Topic** | **Item #** | **Checklist item** | **Location where item is reported** |
| --- | --- | --- | --- |
| **TITLE** | | | Page: |
| Title | 1 | Identify the report as a systematic review. | 1 |
| **ABSTRACT** | | |  |
| Abstract | 2 | See the PRISMA 2020 for Abstracts checklist. | See the abstract checklist |
| **INTRODUCTION** | | |  |
| Rationale | 3 | Describe the rationale for the review in the context of existing knowledge. | 3, 4 |
| Objectives | 4 | Provide an explicit statement of the objective(s) or question(s) the review addresses. | 4 |
| **METHODS** | | |  |
| Eligibility criteria | 5 | Specify the inclusion and exclusion criteria for the review and how studies were grouped for the syntheses. | 5 & Appendix 2 |
| Information sources | 6 | Specify all databases, registers, websites, organisations, reference lists and other sources searched or consulted to identify studies. Specify the date when each source was last searched or consulted. | 5 & Appendix 3 |
| Search strategy | 7 | Present the full search strategies for all databases, registers and websites, including any filters and limits used. | 5 & Appendix 3 |
| Selection process | 8 | Specify the methods used to decide whether a study met the inclusion criteria of the review, including how many reviewers screened each record and each report retrieved, whether they worked independently, and if applicable, details of automation tools used in the process. | 6 |
| Data collection process | 9 | Specify the methods used to collect data from reports, including how many reviewers collected data from each report, whether they worked independently, any processes for obtaining or confirming data from study investigators, and if applicable, details of automation tools used in the process. | 6 |
| Data items | 10a | List and define all outcomes for which data were sought. Specify whether all results that were compatible with each outcome domain in each study were sought (e.g. for all measures, time points, analyses), and if not, the methods used to decide which results to collect. | 6 |
|  | 10b | List and define all other variables for which data were sought (e.g. participant and intervention characteristics, funding sources). Describe any assumptions made about any missing or unclear information. | 6 |
| Study risk of bias assessment | 11 | Specify the methods used to assess risk of bias in the included studies, including details of the tool(s) used, how many reviewers assessed each study and whether they worked independently, and if applicable, details of automation tools used in the process. | 6 & Appendix 5 |
| Effect measures | 12 | Specify for each outcome the effect measure(s) (e.g. risk ratio, mean difference) used in the synthesis or presentation of results. | NA |
| Synthesis methods | 13a | Describe the processes used to decide which studies were eligible for each synthesis (e.g. tabulating the study intervention characteristics and comparing against the planned groups for each synthesis (item #5)). | 6, 7 |
|  | 13b | Describe any methods required to prepare the data for presentation or synthesis, such as handling of missing summary statistics, or data conversions. | 6, 7 |
|  | 13c | Describe any methods used to tabulate or visually display results of individual studies and syntheses. | 6, 7 |
|  | 13d | Describe any methods used to synthesize results and provide a rationale for the choice(s). If meta-analysis was performed, describe the model(s), method(s) to identify the presence and extent of statistical heterogeneity, and software package(s) used. | NA |
|  | 13e | Describe any methods used to explore possible causes of heterogeneity among study results (e.g. subgroup analysis, meta-regression). | NA |
|  | 13f | Describe any sensitivity analyses conducted to assess robustness of the synthesized results. | NA |
| Reporting bias assessment | 14 | Describe any methods used to assess risk of bias due to missing results in a synthesis (arising from reporting biases). | NA |
| Certainty assessment | 15 | Describe any methods used to assess certainty (or confidence) in the body of evidence for an outcome. | NA |
| **RESULTS** | | |  |
| Study selection | 16a | Describe the results of the search and selection process, from the number of records identified in the search to the number of studies included in the review, ideally using a flow diagram. | 8 & 9 (Figure 1) |
|  | 16b | Cite studies that might appear to meet the inclusion criteria, but which were excluded, and explain why they were excluded. | 6, Appendix 4 |
| Study characteristics | 17 | Cite each included study and present its characteristics. | 8, 23 – 30 (Table 1), Appendix 6 |
| Risk of bias in studies | 18 | Present assessments of risk of bias for each included study. | 10 & Appendix 7 |
| Results of individual studies | 19 | For all outcomes, present, for each study: (a) summary statistics for each group (where appropriate) and (b) an effect estimate and its precision (e.g. confidence/credible interval), ideally using structured tables or plots. | 31 – 35 (Table 2), Appendix 8 |
| Results of syntheses | 20a | For each synthesis, briefly summarise the characteristics and risk of bias among contributing studies. | Qualitative synthesis:  10 – 17 |
|  | 20b | Present results of all statistical syntheses conducted. If meta-analysis was done, present for each the summary estimate and its precision (e.g. confidence/credible interval) and measures of statistical heterogeneity. If comparing groups, describe the direction of the effect. | NA |
|  | 20c | Present results of all investigations of possible causes of heterogeneity among study results. | NA |
|  | 20d | Present results of all sensitivity analyses conducted to assess the robustness of the synthesized results. | NA |
| Reporting biases | 21 | Present assessments of risk of bias due to missing results (arising from reporting biases) for each synthesis assessed. | NA |
| Certainty of evidence | 22 | Present assessments of certainty (or confidence) in the body of evidence for each outcome assessed. | NA |
| **DISCUSSION** | | |  |
| Discussion | 23a | Provide a general interpretation of the results in the context of other evidence. | 18 – 20 |
|  | 23b | Discuss any limitations of the evidence included in the review. | 21 |
|  | 23c | Discuss any limitations of the review processes used. | 21 |
|  | 23d | Discuss implications of the results for practice, policy, and future research. | 20 |
| **OTHER INFORMATION** | | |  |
| Registration and protocol | 24a | Provide registration information for the review, including register name and registration number, or state that the review was not registered. | 5 |
|  | 24b | Indicate where the review protocol can be accessed, or state that a protocol was not prepared. | NA |
|  | 24c | Describe and explain any amendments to information provided at registration or in the protocol. | NA |
| Support | 25 | Describe sources of financial or non-financial support for the review, and the role of the funders or sponsors in the review. | 22 |
| Competing interests | 26 | Declare any competing interests of review authors. | 22 |
| Availability of data, code and other materials | 27 | Report which of the following are publicly available and where they can be found: template data collection forms; data extracted from included studies; data used for all analyses; analytic code; any other materials used in the review. | NA |

Table S1.2 PRISMA abstract checklist

| **Section and Topic** | **Item #** | **Checklist item** | **Reported (Yes/No)** |
| --- | --- | --- | --- |
| **TITLE** | | |  |
| Title | 1 | Identify the report as a systematic review. | Yes |
| **BACKGROUND** | | |  |
| Objectives | 2 | Provide an explicit statement of the main objective(s) or question(s) the review addresses. | Yes |
| **METHODS** | | |  |
| Eligibility criteria | 3 | Specify the inclusion and exclusion criteria for the review. | No |
| Information sources | 4 | Specify the information sources (e.g. databases, registers) used to identify studies and the date when each was last searched. | Yes |
| Risk of bias | 5 | Specify the methods used to assess risk of bias in the included studies. | No |
| Synthesis of results | 6 | Specify the methods used to present and synthesise results. | Yes |
| **RESULTS** | | |  |
| Included studies | 7 | Give the total number of included studies and participants and summarise relevant characteristics of studies. | Yes |
| Synthesis of results | 8 | Present results for main outcomes, preferably indicating the number of included studies and participants for each. If meta-analysis was done, report the summary estimate and confidence/credible interval. If comparing groups, indicate the direction of the effect (i.e. which group is favoured). | Yes |
| **DISCUSSION** | | |  |
| Limitations of evidence | 9 | Provide a brief summary of the limitations of the evidence included in the review (e.g. study risk of bias, inconsistency and imprecision). | No |
| Interpretation | 10 | Provide a general interpretation of the results and important implications. | Yes |
| **OTHER** | | |  |
| Funding | 11 | Specify the primary source of funding for the review. | No |
| Registration | 12 | Provide the register name and registration number. | No |

# Appendix 2 Eligibility criteria

Table S2 Inclusion and exclusion criteria for study selection

| Eligible criteria | | Details |
| --- | --- | --- |
| Inclusion criteria | |  |
|  | Population of interest | We included all healthcare providers regardless of their profession or disciplines, as well as the general public, including but not limited to individuals with LTBI, contacts of TB patients, TB patients, patients with other co-morbidities, healthcare or non-healthcare students, immigrants or healthy adults. |
|  | Condition to be studied | LTBI, regardless of the stages in the LTBI cascade of care. This includes the contact tracing process, LTBI screening, LTBI diagnosis, LTBI treatment recommendation, LTBI treatment initiation, LTBI treatment completion and follow-up monitoring. |
|  | Outcomes to be synthesized | The barriers to LTBI management based on the KAP categories. The Knowledge (K) category includes but not limited to the understanding of LTBI clinical characteristics, LTBI screening, LTBI treatment and BCG vaccination. The Attitude (A) category includes but not limited to the acceptability and concerns to LTBI screening and treatment. The Practice (P) category includes the experience of healthcare providers in LTBI screening and treatment. |
|  | Study designs | Observational studies which include descriptive-survey research or cross-sectional study utilizing KAP survey or questionnaires. In the cases where mixed methods studies were screened, only the survey-based section of the studies would be extracted for analysis. |
| Exclusion criteria | | Studies were excluded if they reported on active pulmonary or extrapulmonary TB disease, multidrug-resistant TB, case report, conference proceeding, study protocol, reviews, and economic analysis. |

LTBI: latent tuberculosis infection; TB: tuberculosis: BCG: Bacillus Calmette–Guérin; KAP: Knowledge, Attitude, Practice

# Appendix 3 Search strategy

Table S3 Search strategy

| Database | # | Search terms |
| --- | --- | --- |
| Pubmed | 56 | #7 AND #16 AND #49 AND #55 |
|  | 55 | #50 OR #51 OR #52 OR #53 OR #54 |
|  | 54 | cross-sectional study[MeSH Terms] |
|  | 53 | descriptive study |
|  | 52 | observational study |
|  | 51 | questionnaire[MeSH Terms] |
|  | 50 | survey[MeSH Terms] |
|  | 49 | #17 OR #18 OR #19 OR #20 OR #21 OR #22 OR #23 OR #24 OR #25 OR #26 OR #27 OR #28 OR #29 OR #30 OR #31 OR #32 OR #33 OR #34 OR #35 OR #36 OR #37 OR #38 OR #39 OR #40 OR #41 OR #42 OR #43 OR #44 OR #45 OR #46 OR #47 OR #48 |
|  | 48 | HIV[MeSH Terms] |
|  | 47 | immunocompromised[MeSH Terms] |
|  | 46 | foreign-born |
|  | 45 | immigrant[MeSH Terms] |
|  | 44 | household contact |
|  | 43 | close contact |
|  | 42 | patient[MeSH Terms] |
|  | 41 | communit*[MeSH Terms] |
|  | 40 | general public |
|  | 39 | medical student[MeSH Terms] |
|  | 38 | community health worker[MeSH Terms] |
|  | 37 | “health care professional*” |
|  | 36 | “health care worker*” |
|  | 35 | public health officer |
|  | 34 | “tuberculosis officer" |
|  | 33 | allied health personnel[MeSH Terms] |
|  | 32 | laboratory personnel[MeSH Terms] |
|  | 31 | hospital staff[MeSH Terms] |
|  | 30 | medical assistant[MeSH Terms] |
|  | 29 | nurse[MeSH Terms] |
|  | 28 | pharmacist[MeSH Terms] |
|  | 27 | medical intern |
|  | 26 | health care student[MeSH Terms] |
|  | 25 | clinician |
|  | 24 | doctor[MeSH Terms] |
|  | 23 | general practitioner[MeSH Terms] |
|  | 22 | resident |
|  | 21 | medical officer |
|  | 20 | physician[MeSH Terms] |
|  | 19 | specialist[MeSH Terms] |
|  | 18 | consultant[MeSH Terms] |
|  | 17 | health care provider[MeSH Terms] |
|  | 16 | #8 OR #9 OR #10 OR #11 OR #12 OR #13 OR #14 OR #15 |
|  | 15 | practice |
|  | 14 | stigma[MeSH Terms] |
|  | 13 | awareness[MeSH Terms] |
|  | 12 | belief[MeSH Terms] |
|  | 11 | perception[MeSH Terms] |
|  | 10 | understanding[MeSH Terms] |
|  | 9 | attitude[MeSH Terms] |
|  | 8 | knowledge [MeSH Terms] |
|  | 7 | #1 OR #2 OR #3 OR #4 OR #5 OR #6 |
|  | 6 | tuberc* |
|  | 5 | tuberculosis prevent* |
|  | 4 | inactive tuberc* |
|  | 3 | latent tuberc* |
|  | 2 | LTBI |
|  | 1 | infection, latent tuberculosis [MeSH Terms] |
|  |  |  |
| Embase (via Ovid) | 1 | tuberculosis/ or latent tuberculosis/ |
|  | 2 | knowledge/ or professional knowledge/ |
|  | 3 | social worker attitude/ or attitude to health/ or health personnel attitude/ or patient attitude/ or physician attitude/ or attitude/ or pharmacist attitude/ |
|  | 4 | risk perception/ or perception/ |
|  | 5 | attitude to health/ or human/ or health belief/ |
|  | 6 | awareness/ |
|  | 7 | stigma/ or social stigma/ |
|  | 8 | general practice/ or prescribing practice/ or clinical practice/ or practice guideline/ or medical practice/ or practice.mp. |
|  | 9 | 2 or 3 or 4 or 5 or 6 or 7 or 8 |
|  | 10 | health care provider.mp. or health care personnel/ |
|  | 11 | consultant.mp. or consultation/ |
|  | 12 | specialist.mp. or medical specialist/ |
|  | 13 | hospital physician/ or physician/ |
|  | 14 | medical officer.mp. or medical personnel/ |
|  | 15 | resident/ |
|  | 16 | general practitioner.mp. or general practitioner/ |
|  | 17 | doctor.mp. or physician/ |
|  | 18 | clinician/ |
|  | 19 | health student/ |
|  | 20 | medical student/ |
|  | 21 | clinical pharmacist/ or pharmacist attitude/ or pharmacist/ or hospital pharmacist/ or community pharmacist/ |
|  | 22 | nurse/ |
|  | 23 | medical assistant/ |
|  | 24 | hospital personnel/ |
|  | 25 | laboratory personnel/ |
|  | 26 | paramedical personnel/ |
|  | 27 | health auxiliary/ |
|  | 28 | general public.mp. |
|  | 29 | community/ or community care/ |
|  | 30 | patient/ |
|  | 31 | household/ |
|  | 32 | immigrant/ |
|  | 33 | foreigner/ |
|  | 34 | immunocompromised patient/ |
|  | 35 | Human immunodeficiency virus/ |
|  | 36 | 10 or 11 or 12 or 13 or 14 or 15 or 16 or 17 or 18 or 19 or 20 or 21 or 22 or 23 or 24 or 25 or 26 or 27 or 28 or 29 or 30 or 31 or 32 or 33 or 34 or 35 |
|  | 37 | survey.mp. |
|  | 38 | questionnaire/ |
|  | 39 | observational study/ |
|  | 40 | cross-sectional study/ |
|  | 41 | 37 or 38 or 39 or 40 |
|  | 42 | 1 and 9 and 36 and 41 |
|  |  |  |
| CINAHL Plus | S49 | S5 AND S14 AND S42 AND S48 |
|  | S48 | S43 OR S44 OR S45 OR S46 OR S47 |
|  | S47 | cross-sectional study |
|  | S46 | descriptive study |
|  | S45 | observational study |
|  | S44 | questionnaire |
|  | S43 | survey |
|  | S42 | S15 OR S16 OR S17 OR S18 OR S19 OR S20 OR S21 OR S22 OR S23 OR S24 OR S25 OR S26 OR S27 OR S28 OR S29 OR S30 OR S31 OR S32 OR S33 OR S34 OR S35 OR S36 OR S37 OR S38 OR S39 OR S40 OR S41 |
|  | S41 | HIV |
|  | S40 | Immunocompromised or immunosuppressed |
|  | S39 | foreign-born |
|  | S38 | Immigrant |
|  | S37 | household contact |
|  | S36 | close contact |
|  | S35 | patient |
|  | S34 | communit* |
|  | S33 | general public |
|  | S32 | lay health worker |
|  | S31 | medical assistant |
|  | S30 | community based health worker |
|  | S29 | community health worker |
|  | S28 | allied health personnel |
|  | S27 | nursing staff |
|  | S26 | hospital staff |
|  | S25 | healthcare students |
|  | S24 | health care students |
|  | S23 | medical interns |
|  | S22 | medical workers |
|  | S21 | nurse |
|  | S20 | physician |
|  | S19 | doctor |
|  | S18 | professionals |
|  | S17 | health care professionals |
|  | S16 | health care workers |
|  | S15 | health care providers |
|  | S14 | S6 OR S7 OR S8 OR S9 OR S10 OR S10 OR S11 OR S12 OR S13 |
|  | S13 | practice |
|  | S12 | stigma |
|  | S11 | awareness |
|  | S10 | belief |
|  | S9 | perception |
|  | S8 | understanding |
|  | S7 | attitude |
|  | S6 | knowledge |
|  | S5 | S1 OR S2 OR S3 OR S4 |
|  | S4 | tuberculosis |
|  | S3 | tuberculosis prevention |
|  | S2 | inactive tuberc* |
|  | S1 | latent tuberculosis |
|  |  |  |
| Web of Science  (WOS) | 5 | #4 AND #3 AND #2 AND #1 |
|  | 4 | ALL = (survey OR questionnaire OR observational study OR descriptive study OR cross-sectional study OR interview OR focus group discussion) |
|  | 3 | ALL = (health care provider* OR health care professional* OR health care worker* OR consultant OR specialist OR physician OR medical officer OR resident OR general practitioner OR doctor OR clinician OR health care student OR medical intern OR pharmacist OR nurse OR medical assistant OR hospital staff OR laboratory personnel OR allied health personnel OR “tuberculosis officer" OR public health officer OR community health worker OR medical student OR general public OR communit* OR patient OR close contact OR household contact OR immigrant OR foreign-born OR immunocompromised OR HIV) |
|  | 2 | ALL = (knowledge OR attitude OR understanding OR perception OR belief OR awareness OR stigma OR practice) |
|  | 1 | ALL= (“latent tuberculosis” OR LTBI OR latent tuberc* OR inactive tuberc* OR tuberculosis prevent* OR tuberc*) |
| ProQuest |  | Anywhere except full text (NOFT): latent tuberculosis OR latent tuberc* OR inactive tuberc* OR tuberculosis prevent* OR tuberc*  **AND**  Anywhere except full text (NOFT): knowledge OR attitude OR understanding OR perception OR belief OR awareness OR stigma OR practice  **AND**  Anywhere except full text (NOFT): survey OR questionnaire OR observational study OR descriptive study OR cross-sectional study  **AND**  Anywhere except full text (NOFT): health care provider* OR health care professional* OR health care worker* OR consultant OR specialist OR physician OR medical officer OR resident OR general practitioner OR doctor OR clinician OR health care student OR medical intern OR pharmacist OR nurse OR medical assistant OR hospital staff OR laboratory personnel OR allied health personnel OR “tuberculosis officer" OR public health officer OR community health worker OR medical student OR general public OR communit* OR patient OR close contact OR household contact OR immigrant OR foreign-born OR immunocompromised OR HIV  **AND** |
|  |  | Source type: Limit to Dissertations & Theses |

# Appendix 4 List of excluded studies

Reasons of exclusion:

- 1. Condition: Study on knowledge and/or attitude, but not focusing on latent tuberculosis infection (LTBI): 9
  2. Outcome: Study on LTBI, but not assessing the knowledge and/or attitude: 4
  3. Detailed survey questions and responses not available: 3
  4. Study design: Qualitative study: 10
  5. Abstract only, full text unavailable: 5
  6. Not English: 1

Table S4 List of excluded studies with reasons

| **Study** | **Reasons of exclusion** | **Category** |
| --- | --- | --- |
| Akande 2020 | Study focusing on knowledge of pulmonary TB and TB disease, but not LTBI | 1 |
| Almohammadi 2019 | Detailed survey questions and responses on knowledge of TB were not presented, only as total mean score | 3 |
| Berrocal-almanza 2019 | Qualitative study | 4 |
| Dobler 2018 | Qualitative study | 4 |
| Dy 2014 | Abstract only, full text unavailable | 5 |
| Gao 2018 | Study evaluated acceptability of LTBI educational intervention, but not on knowledge and/or attitude | 2 |
| Goswami 2012 | Study evaluated predictors to LTBI treatment initiation and completion, but not on knowledge and/or attitude | 2 |
| Huddart 2018 | Study focusing on knowledge of TB disease, but not LTBI | 1 |
| Ikram 2019 | Abstract only, full text unavailable | 5 |
| Janagond 2017 | Detailed survey questions and responses on knowledge of TB prevention and control were not presented, only as scores: 0-5, 6-10, >10 | 3 |
| Joseph 2004 | Qualitative Study | 4 |
| Kerrigan 2018 | Qualitative Study | 4 |
| Lai 2019 | Study investigated factors associated with IPT uptake rates, not focusing on knowledge and/or attitude towards IPT | 2 |
| MacDonald 2010 | Qualitative Study | 4 |
| Manabe 2019 | Abstract only, full text unavailable | 5 |
| McEwen 2005 | Qualitative Study | 4 |
| Milinkovic 2019 | Qualitative Study | 4 |
| Naureckas 2015 | Questions and responses focused on testing for HIV in LTBI patients; no question on LTBI asked | 1 |
| Ngo 2019 | Study focused on knowledge of TB disease and prevalence of LTBI, but not knowledge and/or attitude of LTBI | 1* |
| O’Brien 2019 | Abstract only, full text unavailable | 5 |
| Rebeiro 2020 | Detailed question and responses (on LTBI treatment acceptance) not available | 3 |
| Rennie 2019 | Study focused on knowledge of TB disease and HIV, but not LTBI | 1 |
| Royce 2017 | Qualitative Study | 4 |
| Salame 2017 | Study focusing on knowledge and beliefs of TB disease, but not LTBI | 1 |
| Semá Baltazar-2020 | Study focused on knowledge and awareness of TB disease and HIV, not LTBI | 1 |
| Tavares 2019 | Study focused on perspectives of healthcare providers on TB treatment, not specifying whether it was treatment for TB disease or preventive therapy for LTBI | 1 |
| Van Rie 2013 | Study outcomes were prevalence of LTBI, but not knowledge and attitude | 2 |
| Wieland 2012 | Qualitative Study | 4 |
| Wilches-Luna 2016 | Not in English | 6 |
| Wilson 2019 (P206) | Abstract only, full text unavailable | 5 |
| Zeladita-Huaman 2021 | Study focused on knowledge of TB disease and health delivery service for IPT | 1 |
| Zelnick 2016 | Qualitative Study | 4 |

TB: tuberculosis; LTBI: latent tuberculosis infection; IPT: isoniazid preventive therapy; HIV: human immunodeficiency virus

*Not fulfilling both Category 1 and 2

# Appendix 5 Risk of bias assessment checklist

To evaluate the risk of bias of the included studies, the checklist developed by Hoy and colleagues was adapted and modified accordingly. We included attrition bias, to assess the possibility of potential bias due to incomplete responses in the pool of submitted surveys. If more than 10.0% of the data were incomplete, risk of bias could arise in the data analysis process [1, 2]. Therefore, we aimed for 90.0% of completeness in the returned surveys. Signalling question on length of the shortest prevalence period was removed, as it was not relevant to KAP surveys, as shown in Table S4.[3]

Since there was no pre-identified scoring system for the checklist, the evaluation process for the risk of bias assessment was conducted as follows:

Each question is annotated with “Yes”, “No”, “Unclear” or “Not applicable”. One point was allocated for every response of “No”, “Unclear” or “Not applicable”, while zero point was allocated for every response with a “Yes”. An individual study with a total score of 0 to 3 was subjected to the “Low Risk of Bias” category; an individual study with a total score of 4 to 6 fell under the “Moderate Risk of Bias” category; and an individual study with a total score of 7 to 10 was classified as the “High Risk of Bias” category.

Table S5: Risk of bias checklist

| Risk of bias item | | Answer:  Yes (Low Risk) = 0  No (High Risk) = 1  Unclear = 1 |
| --- | --- | --- |
| External validity | |  |
| 1. Was the study’s target sample a close representation of the targeted population in relation to relevant variables, for example, age, sex? | |  |
| 2. Was the sampling frame a true or close representation of the target population? | |  |
| 3. Was some form of random selection used to select the sample, OR, was a census undertaken? | |  |
| 4. Was the likelihood of non-response bias minimal, with a response rate of at least 80%? | |  |
| Internal validity | |  |
| 5. Were data collected directly from the subjects (as opposed to a proxy)? | |  |
| 6. Was acceptable case definition or description of latent tuberculosis infection (LTBI) used in the study? | |  |
| 7. Was a reliable and validated study instrument used to evaluate the knowledge, attitude or practice of latent tuberculosis infection (LTBI) among the study participants? | |  |
| 8. Was the same mode of data collection used for all subjects? | |  |
| 9. Were the numerator(s) and denominator(s) for the  parameter of interest appropriate? | |  |
| 10. Was the survey questionnaire complete without any missing data, OR, more than 90% complete? [1, 2] | |  |
| 11. Summary item on the overall risk of study bias | |  |
| LOW RISK OF BIAS  MODERATE RISK OF BIAS  HIGH RISK OF BIAS | 0-3  4-6  7-10 |  |

# Appendix 6 Summary of study characteristics

Table S6 Summary of study characteristics

| **Characteristics** | | **Number of studies** |
| --- | --- | --- |
| **Targeted respondents:** | **Healthcare providers** |  |
|  | Doctors only | 18 [4-21] |
|  | Doctors and nurses / nursing staff | 6 [22-27] |
|  | Doctors, nurses, laboratory staff and pharmacists | 1 [28] |
|  | Community healthcare workers and nurse aides | 1 [29] |
|  | Doctors, nurses and other health care staff including administrators with medical background and research scientists | 1 [30] |
|  | Nursing, administrative / clerical, medical staff, research scientist, lab technician, and therapist | 1 [31] |
|  | Health care students | 3 [32-34] |
|  | Not specified | 1 [35] |
| **Targeted respondents:** | **Public level** |  |
|  | Individuals eligible for LTBI treatment | 13 [36-46] [30] [43] |
|  | Individuals from high TB burden countries | 2 [47, 48] |
|  | TB patients | 1 [49] |
|  | Caregiver to children eligible for LTBI treatment | 1 [50] |
| **Healthcare providers’ discipline** | Rheumatological, gastroenterological and dermatological departments | 6 [6, 9, 14, 16, 18, 20] |
|  | Paediatrics | 4 [7, 13, 17, 22] |
|  | PLHIV | 2 [8, 24] |
| **KAP Category** | Knowledge (K) | 4 [28, 36, 38, 42] |
|  | Attitude (A) | 4 [21, 30, 35, 46] |
|  | Practice (P) | 7 [6-9, 13, 20, 33] |
|  | Knowledge (K) and Attitude (A) | 17 [5, 11, 15, 23, 25, 27, 31, 37, 39-41, 44, 45, 47-50] |
|  | Knowledge (K) and Practice (P) | 6 [12, 14, 16, 17, 32, 34] |
|  | Attitude (A) and Practice (P) | 4 [4, 10, 18, 19] |
|  | Knowledge (K), Attitude (A) and Practice (P) | 5 [22, 24, 26, 29, 43] |

LTBI: latent tuberculosis infection; TB: tuberculosis; PLHIV: people living with human immunodeficiency virus

# Appendix 7 Risk of bias of the included studies

Table S7 Risk of bias evaluation for included studies

| Study | 1 | 2 | 3 | 4 | 5 | 6 | 7 | 8 | 9 | 10 | Score | Overall risk of bias |
| --- | --- | --- | --- | --- | --- | --- | --- | --- | --- | --- | --- | --- |
| Ailinger 2004 [36] | Y | U | N | Y | Y | Y | Y | Y | Y | Y | 2 | L |
| Alotaibi 2019 [28] | Y | Y | Y | Y | Y | Y | Y | Y | Y | Y | 0 | L |
| Atchison 2015 [4] | Y | Y | Y | Y | Y | Y | Y | Y | Y | Y | 0 | L |
| Bhanot 2012 [5] | Y | Y | Y | Y | Y | Y | U | Y | Y | Y | 1 | L |
| Biedenharn 2015 [37] | Y | Y | N | Y | Y | Y | U | Y | Y | Y | 2 | L |
| Butcher 2013 [38] | Y | Y | N | Y | Y | Y | Y | Y | Y | Y | 1 | L |
| Cantini 2016 [6] | Y | Y | Y | Y | Y | Y | U | Y | Y | Y | 1 | L |
| Chiang 2015 [22] | Y | Y | Y | Y | Y | Y | Y | Y | Y | Y | 0 | L |
| Colson 2010 [39] | Y | Y | Y | Y | Y | Y | U | Y | Y | Y | 1 | L |
| Colson 2013 [40] | Y | Y | Y | Y | Y | Y | U | Y | Y | Y | 1 | L |
| Coreil 2012 [30] | Y | Y | N | Y | Y | Y | Y | Y | Y | Y | 1 | L |
| Cruz 2016 [7] | N | U | Y | N | Y | Y | Y | Y | Y | N | 4 | M |
| DeLuca 2018 [41] | Y | U | N | Y | Y | Y | Y | Y | Y | Y | 2 | L |
| Evenblij 2016 [8] | N | U | N | N | Y | Y | U | Y | Y | Y | 5 | M |
| Gao 2015 [42] | Y | Y | N | Y | Y | Y | Y | Y | Y | Y | 1 | L |
| Gupta 2011 [9] | N | U | Y | N | Y | Y | U | Y | Y | Y | 4 | M |
| Gutsfeld 2014 [10] | N | U | Y | N | Y | Y | Y | Y | Y | Y | 3 | L |
| Hill 2010 [43] | Y | U | N | Y | Y | Y | U | Y | Y | Y | 3 | L |
| Hirsch-Moverman 2006 [11] | Y | U | Y | Y | Y | Y | U | Y | Y | Y | 2 | L |
| Hirsch-Moverman 2013 [31] | Y | Y | Y | Y | Y | Y | U | Y | Y | Y | 1 | L |
| Howley 2015 [49] | N | Y | N | N | Y | Y | U | Y | Y | Y | 4 | M |
| Jackson 2007 [32] | N | U | Y | N | Y | Y | U | Y | Y | Y | 4 | M |
| Kane 2013 [44] | Y | U | N | Y | Y | Y | U | Y | Y | Y | 3 | L |
| Karakousis 2007 [12] | N | Y | Y | N | Y | Y | Y | Y | Y | Y | 2 | L |
| Lazar 2010 [13] | N | U | Y | N | Y | Y | Y | Y | Y | N | 4 | M |
| Li 2018 [45] | Y | U | Y | Y | Y | Y | U | Y | Y | Y | 2 | L |
| Mirtskhulava 2015 [23] | Y | Y | N | Y | Y | Y | Y | Y | Y | Y | 1 | L |
| Montagna 2014 [34] | Y | U | N | Y | Y | Y | Y | Y | Y | Y | 2 | L |
| Montagna 2018 [33] | Y | U | N | Y | Y | Y | Y | Y | Y | Y | 2 | L |
| Moolphate 2013 [24] | N | U | Y | N | Y | Y | Y | Y | Y | N | 4 | M |
| Narayanan 2019 [47] | Y | U | N | Y | Y | Y | Y | Y | Y | Y | 2 | L |
| O’Donnell 2011 [46] | Y | U | N | Y | Y | Y | U | Y | Y | Y | 3 | L |
| Pathak 2016 [25] | N | U | Y | N | Y | Y | U | Y | Y | Y | 4 | M |
| Quirós 2018 [14] | Y | Y | N | Y | Y | Y | U | Y | Y | Y | 2 | L |
| Ramos 2018 [26] | Y | U | N | Y | Y | Y | Y | Y | Y | Y | 2 | L |
| Salazar-Schicchi 2004 [15] | Y | Y | Y | Y | Y | Y | Y | Y | Y | Y | 0 | L |
| Skinner 2013 [50] | Y | U | N | Y | Y | Y | U | Y | Y | Y | 3 | L |
| Smith 2012 [16] | N | U | Y | N | Y | Y | Y | Y | Y | Y | 3 | L |
| Spruijt 2020 [27] | Y | U | Y | Y | Y | Y | U | Y | Y | Y | 2 | L |
| Stout 2006 [17] | N | U | Y | N | Y | Y | U | Y | Y | Y | 4 | M |
| Trajman 2019 [29] | Y | Y | N | Y | Y | Y | Y | Y | Y | Y | 1 | L |
| Tran 2017 [18] | N | U | Y | N | Y | Y | U | Y | Y | Y | 4 | M |
| Vinnard 2012 [19] | N | U | N | N | Y | Y | Y | Y | Y | N | 5 | M |
| Walker 2018 [48] | N | U | Y | N | Y | Y | Y | Y | Y | N | 4 | M |
| Xerinda 2016 [20] | N | Y | Y | N | Y | Y | Y | Y | Y | Y | 2 | L |
| Yates 2015 [21] | Y | U | Y | Y | Y | Y | U | Y | Y | Y | 2 | L |
| Zhou 2014 [35] | Y | U | Y | Y | Y | Y | U | Y | Y | Y | 2 | L |

Y: Yes: U: Unclear; N: No

# Appendix 8 Findings of included studies by KAP category, level (general public, provider, system), and sub-component (COM-B)

Table S8 Findings of included studies by KAP category, level (G: general public, Pr: provider, S: system), and sub-component (COM-B)

*Heading abbreviations: KAP = knowledge, attitude, practice; COM-B = capability, opportunity, motivation, behaviour; K = knowledge; A = attitude; P = practice; G = general public; Pr = provider; S = system; PhC = physical capability; PsC = psychological capability; RM = reflective motivation; AM = automatic motivation; PO = physical opportunity; SO = social opportunity*

| Study | Response to corresponding survey questions | KAP Category | | | Level | | | COM-B subcomponent | | | | | |
| --- | --- | --- | --- | --- | --- | --- | --- | --- | --- | --- | --- | --- | --- |
|  |  | K | A | P | G | Pr | S | PhC | PsC | RM | AM | PO | SO |
| Ailinger 2004 [36] | The mean score on the TB Knowledge Instrument was 66% with a range of 20–93% and an SD of 17. | 🗸 |  |  | 🗸 |  |  |  | 🗸 |  |  |  |  |
|  | ‘If you have been vaccinated with BCG, you cannot develop TB’’ received the most ‘‘Don’t Know’’ responses (46%). | 🗸 |  |  | 🗸 |  |  |  | 🗸 |  |  |  |  |
|  | The numbness and tingling in the hands as a side effect of INH therapy was one of questions most frequently answered incorrectly (53%). | 🗸 |  |  | 🗸 |  |  |  | 🗸 |  |  |  |  |
| Alotaibi 2019 [28] | Knowledge about LTBI screening test:  Did not know: 7.24%  TST: 59.96%  IGRA: 12.73%  Sputum culture: 44.35%  Sputum acid-fast bacilli smear test: 35.32%  Chest X-ray: 52.98% | 🗸 |  |  |  | 🗸 |  |  | 🗸 |  |  |  |  |
|  | Patients with LTBI can spread the disease.  Knowledge score: 0.33±0.47  (Scoring scale - 0: Incorrect; 1: Correct)  Correct answer:  False: 32.82%  Incorrect answer:  Don’t know: 12.04%  True: 55.15% | 🗸 |  |  |  | 🗸 |  |  | 🗸 |  |  |  |  |
|  | Patients with LTBI have positive reaction on TST/IGRA tests.  Knowledge score: 0.57±0.49  (Scoring scale - 0: Incorrect; 1: Correct)  Correct answer:  True: 57.43%  Incorrect answer:  Don’t know: 31.68%  False: 10.89% | 🗸 |  |  |  | 🗸 |  |  | 🗸 |  |  |  |  |
|  | BCG vaccination is protective against TB.  Don’t know: 27.24%  True: 36.77%  False: 35.99%  The authors found HCWs were unclear on the value of BCG vaccination in relation to TB prevention. | 🗸 |  |  |  | 🗸 |  |  | 🗸 |  |  |  |  |
| Atchison 2015 [4] | LTBI screening and treatment of migrants from high incidence countries was an important strategy for reducing TB incidence in the UK:  76 (68 %; 95 % CI 59 %–76 %) GPs either strongly agreed or agreed;  4 GPs (4 %; 95 % CI 1 %–9 %) disagreed with this statement. |  |  | 🗸 |  |  | 🗸 |  |  | 🗸 |  |  |  |
|  | 33 (29 %; 95 % CI 22 %–39 %) GPs reported previous experience of screening or treatment of patients with active or LTBI; this was predominantly in the context of hospital infectious diseases (n = 7) or respiratory medicine (n = 14) placements as junior doctors, or as part of an overseas post (n = 5). |  |  | 🗸 |  | 🗸 |  | 🗸 |  |  |  |  |  |
|  | 97 (87 %; 95 % CI 79 %–92 %) GPs reported that they had not screened for or treated LTBI as part of their practice as a GP. |  |  | 🗸 |  | 🗸 |  | 🗸 |  |  |  |  |  |
|  | LTBI screening:  73 (65 %; 95 % CI 56 %–74 %) GPs reported they felt confident in ruling out active TB with chest x-ray and clinical examination. |  | 🗸 |  |  | 🗸 |  |  | 🗸 |  |  |  |  |
|  | LTBI treatment:  12 (11 %; 95 % CI 6–18) GPs reported they felt confident in initiating LTBI drug treatment and 9 (8 %; 95 % CI 4–15) GPs reported confidence in adjusting drug doses of individuals on LTBI treatment. |  | 🗸 |  |  | 🗸 |  |  | 🗸 |  |  |  |  |
|  | 62 (64 %; 95 % CI 54 %–73 %) GPs stated that they would refer individuals for LTBI screening and treatment to secondary care. |  |  | 🗸 |  | 🗸 |  | 🗸 |  |  |  |  |  |
|  | There was a need for a primary care-based GP-led service for LTBI treatment for adult migrants:  59 (53 %; 95 % CI 43 %–62 %) GPs either strongly agreed or agreed;  21 (19 %; 95 % CI 12 %–27 %) GPs disagreed with this statement. |  |  | 🗸 |  |  | 🗸 |  |  |  |  | 🗸 |  |
|  | Willing to deliver LTBI treatment in primary care:  6 (5 %; 95 % CI 2 %–12 %) GPs willing without any condition; while 87 (78 %; 95 % CI 69 %–85 %) GPs willing if key barriers were addressed and suggested enablers considered when developing the service model. |  | 🗸 |  |  | 🗸 |  |  |  | 🗸 |  |  |  |
|  | Barrier 1: Insufficient experience among GPs regarding all aspects of LTBI screening and treatment (82 %; 95 % CI 74 %–88 %).  Suggestion: 88 (79 %; 95 % CI 70 %–85 %) GPs suggested having specific training in LTBI screening and treatment. |  |  | 🗸 |  | 🗸 |  | 🗸 |  |  |  |  |  |
|  | Barrier 2: Lack of timely access to specialist TB services and support (61 %; 95 % CI 51 %–69 %).  Suggestion: 63 (56 %; 95 % CI 47 %–65 %) GPs stated easy access and timely support from specialist TB teams would facilitate delivering LTBI treatment in primary care as well as developing clear referral pathways to secondary care for more complex cases. |  |  | 🗸 |  |  | 🗸 |  |  |  |  | 🗸 |  |
|  | Barrier 3: 76(68 %; 95 % CI 59 %–76 %) GPs stated that more resources (in terms of human and logistic resources) would be required.  Suggestion: To have more and longer GP appointments, appointing a GP with TB or respiratory medicine special interest accreditation, appointing a community-based TB nurse, and dedicated phlebotomist and **community pharmacist** sessions. |  |  | 🗸 |  |  | 🗸 |  |  |  |  | 🗸 |  |
|  | Barrier 4: Many GPs did not know or felt unable to comment on the type of funding or financial resources required.  Suggestion: Specific funding / financial incentives was viewed as an important enabler (63 %; 95 % CI 53 %–71 %). Several GPs suggested to have contractual arrangements such as a Locally Enhanced Service (LES) or Quality and Outcomes Framework  (QOF) targets. |  |  | 🗸 |  | 🗸 |  |  |  |  |  | 🗸 |  |
| Bhanot 2012 [5] | 10/38 physicians tested positive for LTBI had initiated therapy for LTBI, 6 had completed entire 9-month of INH. |  | 🗸 |  |  | 🗸 |  |  |  | 🗸 |  |  |  |
|  | 34/46 physician would take IPT if negative PPD status converted to positive. |  | 🗸 |  |  | 🗸 |  |  |  | 🗸 |  |  |  |
|  | Reason for not adhering to LTBI therapy:  Side effects of INH: 24/29 (82.75%) |  | 🗸 |  |  | 🗸 |  |  |  | 🗸 |  |  |  |
|  | Reason for not adhering to LTBI therapy:  Length of therapy: 12/29 (41.37%) |  | 🗸 |  |  | 🗸 |  |  |  | 🗸 |  |  |  |
|  | Reason for not adhering to LTBI therapy:  History of BCG vaccination 12/29 (41.37%) | 🗸 |  |  |  | 🗸 |  |  | 🗸 |  |  |  |  |
| Biedenharn 2015 [37] | LTBI is contagious.  Correct answer:  False: 22 (52.38%) | 🗸 |  |  | 🗸 |  |  |  | 🗸 |  |  |  |  |
|  | LTBI diagnosis:  Positive skin test means you have disease  Correct answer:  False: 28 (66.67%) | 🗸 |  |  | 🗸 |  |  |  | 🗸 |  |  |  |  |
|  | BCG prevents TB disease:  Attitude score: 2.73±0.99  (Scoring scale - 1: Strongly disagree; 4: Strongly agree) | 🗸 |  |  | 🗸 |  |  |  | 🗸 |  |  |  |  |
|  | **Subjective norm**  You are embarrassed to tell people you have the TB germ:  Attitude score: 2.33±1.16  (Scoring scale - 1: Strongly disagree; 4: Strongly agree) |  | 🗸 |  | 🗸 |  |  |  |  |  | 🗸 |  |  |
|  | **Subjective norm**  You care about what family/friends think about LTBI treatment:  Attitude score: 2.69±1.02  (Scoring scale - 1: Strongly disagree; 4: Strongly agree) |  | 🗸 |  | 🗸 |  |  |  |  |  |  |  | 🗸 |
| Butcher 2013 [38] | **Understanding of LTBI and INH treatment**  Why are you coming to this clinic?  **Knew they had LTBI: 41 (89.1%)**  Couldn’t distinguish between TB &  LTBI: 4 (8.7%)  Unsure: 1 (2.2%) | 🗸 |  |  | 🗸 |  |  |  | 🗸 |  |  |  |  |
|  | Does this condition (LTBI) make you sick?  Yes: 2 (4.4%)  **No: 44 (95.7%)** | 🗸 |  |  | 🗸 |  |  |  | 🗸 |  |  |  |  |
|  | Can you give this condition to anyone else?    Yes: 1 (2.3%)  **No: 43 (93.5%)**  Unsure: 2 (4.4%) | 🗸 |  |  | 🗸 |  |  |  | 🗸 |  |  |  |  |
|  | How will taking the tablets help you?  **Reduce the risk of active TB in the future: 41 (89.1%)**  Unsure: 5 (10.9%) | 🗸 |  |  | 🗸 |  |  |  | 🗸 |  |  |  |  |
| Cantini 2016 [6] | Availability of LTBI screening tools:  TST and IGRA were available in 77.9% and 71% of the centers, respectively. |  |  | 🗸 |  |  | 🗸 |  |  |  |  | 🗸 |  |
|  | LTBI screening test:  Chest radiograph (CR): 5%  TST: 5.3%  CR + TST: 35.6%  IGRAs: 7.4%  CR + IGRAs: 26%  CR + TST + IGRAs: 20.6%. |  |  | 🗸 |  |  | 🗸 | 🗸 | 🗸 |  |  |  |  |
|  | TB prevention (LTBI treatment) was initiated:  Positive TST: 97 (24.7%)  Positive TST + IGRAs: 101 (25.7%)  Positive IGRAs: 195 (49.6%) |  |  | 🗸 |  |  | 🗸 | 🗸 | 🗸 |  |  |  |  |
|  | When TB prevention (LTBI treatment) was indicated, anti-TNF therapy was started:  At a 1-month interval in 63.1% of the cases;  After 3 months in 27.7%;  Concomitantly in 5.6%;  After variable intervals ranging from 2 to 9 months in 3.6%. |  |  | 🗸 |  |  | 🗸 | 🗸 | 🗸 |  |  |  |  |
|  | LTBI treatment regimen prescription:  Isoniazid (INH) 9-month course for TB prevention: 324/393 (82.5%);  INH + rifampin for 4 months: 19/393 (4.8%);  Rifampin alone by 6/393 (1.5%);  Other strategies after consultation with the local infectious disease specialist: 44 (11.2%).  **Combined vitamin B6 to prevent INH-induced peripheral neuropathy was always given by 64.9% of clinicians**, sometimes by 13%, while 21%  did not associate the drug. |  |  | 🗸 |  |  | 🗸 | 🗸 | 🗸 |  |  |  |  |
|  | After commencing anti-TNF, LTBI screening was respectively repeated at:  12-month interval: 190 (48.3%);  6-month interval: 70 (17.8%);  18- month interval: 44 (11.2%);  Only in case of clinical suspicion of TB (with TST or IGRA, or both): 89 (22.6%) |  |  | 🗸 |  |  | 🗸 | 🗸 | 🗸 |  |  |  |  |
| Chiang 2015 [22] | What is the definition of latent TB infection?  Correct answer:  When someone has been infected with M. tuberculosis and has a positive TST but has neither symptoms nor abnormal CXR findings: 121/310 (39.0%) | 🗸 |  |  |  | 🗸 |  |  | 🗸 |  |  |  |  |
|  | Compared to adults, children <5 years of age are at higher risk for progression from LTBI to disease?  Correct answer:  True: 200/310 (64.5%) | 🗸 |  |  |  | 🗸 |  |  | 🗸 |  |  |  |  |
|  | Which children are candidates for IPT?  Correct answer:  Asymptomatic children under age 5 who are household contacts of TB patients, regardless of whether the TST is positive or negative AND children under age 15 with positive TST, normal CXR, and no clinical symptoms: 94/310 (30.3%) | 🗸 |  |  |  | 🗸 |  |  | 🗸 |  |  |  |  |
|  | How do you manage a neonate with a normal exam and CXR whose mother is in her second month of treatment for pulmonary TB?  Correct answer:  Initiate IPT: 38/103 (37%) | 🗸 |  |  |  | 🗸 |  |  | 🗸 |  |  |  |  |
|  | What are contra-indications for IPT?  Correct answer:  The child already has TB disease: 258/310 (83.2%) | 🗸 |  |  |  | 🗸 |  |  | 🗸 |  |  |  |  |
|  | Barrier 1: Childhood TB training was not mandated by National Tuberculosis Programme (NTP) policy, infrequent, and sessions were open to a limited number of participants. |  |  | 🗸 |  |  | 🗸 | 🗸 |  |  |  |  |  |
|  | Barrier 2: Providers did not attend training because of competing work responsibilities.  Suggestion: Improving the availability  and quality of educational interventions may help decrease the number of childhood TB cases and deaths. |  | 🗸 |  |  | 🗸 |  |  |  |  |  | 🗸 |  |
| Colson 2010 [39] | LTBI is contagious:  Correct answer:  False: 144 (57.4%) | 🗸 |  |  | 🗸 |  |  |  | 🗸 |  |  |  |  |
|  | PPD-positive means you have TB disease:  Correct answer:  False: 175 (69.7%) | 🗸 |  |  | 🗸 |  |  |  | 🗸 |  |  |  |  |
|  | BCG vaccine prevents TB disease:  Attitude score: 2.95±1.086  (Scoring scale - 1: Strongly disagree; 4: Strongly agree) | 🗸 |  |  | 🗸 |  |  |  | 🗸 |  |  |  |  |
|  | **Stigma**  Embarrassed to have your LTBI status known:  Attitude score: 2.23±1. 183  (Scoring scale - 1: Strongly disagree; 4: Strongly agree) |  | 🗸 |  | 🗸 |  |  |  |  |  | 🗸 |  |  |
|  | **Group norms**  Care about what family and friends think of LTBI treatment:  Attitude score: 2.71±1.204  (Scoring scale - 1: Strongly disagree; 4: Strongly agree) |  | 🗸 |  | 🗸 |  |  |  |  |  |  |  | 🗸 |
|  | Perceived barriers:  You would suffer from side effects  Attitude score: 2.51±0.713  (Scoring scale - 1: Strongly disagree; 4: Strongly agree) |  | 🗸 |  | 🗸 |  |  |  |  |  |  | 🗸 |  |
|  | Perceived barriers:  You do not think you really have “TB germ  Attitude score: 2.22±0.799  (Scoring scale - 1: Strongly disagree; 4: Strongly agree) |  | 🗸 |  | 🗸 |  |  |  |  | 🗸 |  |  |  |
|  | Perceived barriers:  Pills hard to swallow  Attitude score: 2.15±0.739  (Scoring scale - 1: Strongly disagree; 4: Strongly agree) |  | 🗸 |  | 🗸 |  |  |  |  |  |  | 🗸 |  |
|  | Perceived barriers:  Your family and friends will shun  Attitude score: 2.05±0.859  (Scoring scale - 1: Strongly disagree; 4: Strongly agree) |  | 🗸 |  | 🗸 |  |  |  |  |  |  |  | 🗸 |
|  | Perceived barriers:  Life so busy do not have time  Attitude score: 1.73±0.604  (Scoring scale - 1: Strongly disagree; 4: Strongly agree) |  | 🗸 |  | 🗸 |  |  |  |  |  |  | 🗸 |  |
| Colson 2013 [40] | Believe they can spread TB germs:  Correct answer:  No 1153 (65.4%)  Incorrect answer:  Yes 470 (26.7%)  Don’t know 139 (7.9%) | 🗸 |  |  | 🗸 |  |  |  | 🗸 |  |  |  |  |
|  | Reasons for non-acceptance:  Concerns about side effects: 57 (24.5%) |  | 🗸 |  | 🗸 |  |  |  |  |  |  | 🗸 |  |
|  | Reasons for non-acceptance:  Medications do not help/refuse to take medicines: 44 (18.9%) |  | 🗸 |  | 🗸 |  |  |  |  | 🗸 |  |  |  |
|  | Reasons for non-acceptance:  Not at risk of TB/TB is low priority: 33 (14.2%) |  | 🗸 |  | 🗸 |  |  |  |  | 🗸 |  |  |  |
|  | Reasons for non-acceptance:  I don’t have TB or LTBI: 28 (12.0%) |  | 🗸 |  | 🗸 |  |  |  |  | 🗸 |  |  |  |
|  | Reasons for non-acceptance:  I don’t feel sick: 26 (11.2%) |  | 🗸 |  | 🗸 |  |  |  |  | 🗸 |  |  |  |
|  | Reasons for non-acceptance:  Doctor’s advice 24 (10.3%) |  | 🗸 |  | 🗸 |  |  |  |  |  |  | 🗸 |  |
|  | Reasons for non-acceptance:  Length of treatment/fear of being non-adherent: 21 (9.0%) |  | 🗸 |  | 🗸 |  |  |  |  |  |  | 🗸 |  |
|  | Reasons for non-acceptance:  Lack of trust in health care system: 19 (8.2%) |  | 🗸 |  | 🗸 |  |  |  |  | 🗸 |  |  |  |
|  | Reasons for non-acceptance:  I don’t like taking medicines: 15 (6.4%) |  | 🗸 |  | 🗸 |  |  |  |  | 🗸 |  |  |  |
|  | Reasons for non-acceptance:  Had BCG vaccine: 9 (3.9%) | 🗸 |  |  | 🗸 |  |  |  | 🗸 |  |  |  |  |
| Coreil 2012 [30] | Stigma scores for healthcare provider:  **Composite score: 26.6±14.7**  Disclose to neighbors: 2.66±0.76  Disclose to co-workers: 2.63±0.77  Hide problem from others: 2.49±0.80  Disclose to church members: 2.39±0.83  Disclose to friends: 1.73±1.05  Assume have TB disease: 1.63±1.29  Others think worse because Haitian: 1.44±1.33  More embarrassing because Haitian: 1.39±1.33  Assume other health problems: 1.30±1.35  **Feel ashamed: 1.19±1.2**  Problems at work: 1.09±1.15  (Mean scores per item were the average score on a Likert scale of 0 -3, with three representing the highest level of stigma) |  | 🗸 |  |  | 🗸 |  |  |  |  | 🗸 |  | 🗸 |
|  | Stigma scores for patients:  **Composite score: 17.1±9.8**  Disclose to neighbors: 2.47±0.84  Disclose to co-workers: 2.31±0.95  Disclose to church members: 2.16±1.00  Hide problem from others: 1.18±1.37  Disclose to friends: 1.69±1.16  (Mean scores per item were the average score on a Likert scale of 0 -3, with three representing the highest level of stigma) |  | 🗸 |  | 🗸 |  |  |  |  |  |  |  | 🗸 |
|  | Anticipated psychosocial distress by healthcare providers:  Sadness, worry or stress: 21 (48.8%)  Social stigma: 10 (23.3%)  Anxiety regarding family: 6 (14.0%)  Providers were almost five times more likely than patients to identify one or more form of psychosocial distress as probable for someone with LTBI |  | 🗸 |  |  | 🗸 | 🗸 |  |  |  | 🗸 |  | 🗸 |
|  | Anticipated psychosocial distress by patients:  Sadness, worry or stress: 15 (16.7%) |  | 🗸 |  | 🗸 |  |  |  |  |  | 🗸 |  |  |
| Cruz 2016 [7] | Experience with LTBI  No cases annually: 32 (16%)  1–5/year: 99 (50%)  1–5/month: 52 (26%)  5–20/month: 10 (5%)  >20/month: 4 (2%) |  |  | 🗸 |  | 🗸 |  | 🗸 |  |  |  |  |  |
|  | Youngest age at which you routinely use IGRAs:  Do not routinely use IGRAs: 15 (9%)  Routinely used IGRAs in all ages: 12 (7%)  1 year: 9 (6%)  2–3 years: 40 (25%)  4–5 years: 39 (24%)  >5 years: 48 (29%) |  |  | 🗸 |  | 🗸 |  | 🗸 |  |  |  |  |  |
|  | In an immunocompetent school-aged child who has not received the BCG vaccine, is there a TST induration above which you would not obtain an IGRA? (162 respondents)  ≥15 mm: 75 (46%)  ≥20 mm 15 (9%)  Other TST size (10, 25 mm): 6 (4%)  I would obtain an IGRA in all such children, regardless of TST size: 45 (28%)  I do not obtain IGRAs in my practice: 8 (5%)  Unsure: 13 (8%) |  |  | 🗸 |  |  | 🗸 | 🗸 |  |  |  |  |  |
|  | Which test of infection would you use for:  10-year-old US-born child with Crohn’s, on steroids, about to start TNF-a antagonist therapy?  TST only: 15 (9%)  IGRA only: 59 (37%)  Both TST and IGRA: 81 (51%)  Tiered testing (the use of a TST and then performing an IGRA only in children with positive TST results): 5 (3%) |  |  | 🗸 |  |  | 🗸 | 🗸 |  |  |  |  |  |
|  | Which test of infection would you use for: 3-year-old US-born child, mother recently diagnosed with pulmonary TB?  TST only: 99 (61%)  IGRA only: 5 (3%)  Both TST and IGRA: 45 (28%)  Tiered testing: 14 (8%) |  |  | 🗸 |  |  | 🗸 | 🗸 |  |  |  |  |  |
|  | Which test of infection would you use for: 15-year-old US-born child, mother recently diagnosed with pulmonary TB?  TST only: 52 (32%)  IGRA only: 62 (39%)  Both TST and IGRA: 32 (20%)  Tiered testing: 15 (9%) |  |  | 🗸 |  |  | 🗸 | 🗸 |  |  |  |  |  |
|  | Which test of infection would you use for:  1-year-old BCG-vaccinated immigrant from India?  TST only: 75 (46%)  IGRA only: 25 (15%)  Both TST and IGRA: 26 (16%)  Tiered testing: 37 (23%) |  |  | 🗸 |  |  | 🗸 | 🗸 |  |  |  |  |  |
|  | Which test of infection would you use for:  6-year-old BCG-vaccinated immigrant from India?  (162 respondents)  TST only: 14 (9%)  IGRA only: 100 (62%)  Both TST and IGRA: 22 (13%)  Tiered testing: 26 (16%) |  |  | 🗸 |  |  | 🗸 | 🗸 |  |  |  |  |  |
|  | An adolescent with Crohn’s disease requires a TNF-α antagonist and has newly diagnosed tuberculous infection. How long would you treat with antituberculosis medication prior to starting the TNF-α antagonist?  2 weeks: 17 (10%)  1–2 months: 82 (50%)  4 months: 4 (2%)  ≥6 months: 8 (5%)  Start at the same time: 23 (14%)  Unsure: 29 (18%) |  |  | 🗸 |  |  | 🗸 | 🗸 |  |  |  |  |  |
|  | Barrier 1:  Lack of IGRA test availability in public health departments due to cost, interpreting indeterminate results, and more definitive guidance on IGRA use in preschool-aged children. |  |  | 🗸 |  |  | 🗸 |  |  |  |  | 🗸 |  |
|  | Barrier 2: Difficulty in finding guidance on the optimal time during which a child should be on LTBI treatment before the initiation of TNF-a antagonists. Participants noted that guidelines in the infectious disease (vs. rheumatology) literature were difficult to find and appeared inconsistent. |  |  | 🗸 |  |  | 🗸 | 🗸 |  |  |  |  |  |
| DeLuca 2018 [41] | I am afraid whether they may infect others (as a person with LTBI):  Correct answer: LTBI is not contagious  No: 45 (45%)  Incorrect answer:  Yes: 46 (46%)  Uncertain: 9 (9%) | 🗸 |  |  | 🗸 |  |  |  | 🗸 |  |  |  |  |
|  | When asked if participants understood the difference between TB infection and disease, (90%) did not or were unsure of the difference. | 🗸 |  |  | 🗸 |  |  |  | 🗸 |  |  |  |  |
|  | Healthcare providers should be giving me medication for LTBI.  Yes: 73 (73%)  No: 24 (24%)  Uncertain: 2 (2%)  73% of our population willing to take preventive therapy in the absence of good understanding of TB transmission and infection. |  | 🗸 |  | 🗸 |  |  |  |  | 🗸 |  |  |  |
|  | A positive TB skin test means that you  already have disease  Correct answer:  False: 37 (37%)  Incorrect answer:  True: 42 (42%)  Uncertain: 21 (21%) | 🗸 |  |  | 🗸 |  |  |  | 🗸 |  |  |  |  |
|  | To assess readiness for preventive therapy, participants were asked whether they are at a higher risk of progressing to active disease because of their LTBI status. 54 (55%) felt that they are at higher risk. |  | 🗸 |  | 🗸 |  |  |  |  | 🗸 |  |  |  |
|  | 92% participants have never heard of preventive therapy. | 🗸 |  |  | 🗸 |  |  |  | 🗸 |  |  |  |  |
| Evenblij 2016 [8] | Of the 51 respondents, 12 (24 %) intended to screen for LTBI in all SCs as the guideline stipulates. |  |  | 🗸 |  | 🗸 |  |  |  | 🗸 |  |  |  |
|  | Of the HIV physicians who intended to screen for LTBI, less than 25 % would use both screening tests, as stipulated in the guideline. |  |  | 🗸 |  |  | 🗸 |  | 🗸 |  |  |  |  |
|  | HIV-physicians intended to screen more frequently for LTBI in scenarios representing HIV-infected patients  from Liberia (High TB burden country), than Dutch HIV-positive patients, regardless of CD4 cell count  (SC I: 38/51, 75 %, and SC II: 27/51, 53 %, versus SC IV: 15/51, 29 %, and SC V: 23/51, 40 %). |  |  | 🗸 |  | 🗸 |  |  |  | 🗸 |  |  |  |
|  | Although low CD4 count is a risk for progression from LTBI to active TB, respondents from both countries were less likely to be screened for LTBI in this setting (SC II: 27/51, 53 %, SC IV 15/51, 20 %). |  |  | 🗸 |  | 🗸 |  |  |  | 🗸 |  |  |  |
|  | HIV-physicians were less inclined to initiate preventive treatment in the treatment scenarios with an absent IGRA (TC I: 21/51, 41 %) or a negative IGRA (TC IV: 22/51, 43 %). |  |  | 🗸 |  | 🗸 |  |  |  | 🗸 |  |  |  |
|  | When both IGRA and TST were positive (TC III), the majority (39/51, 76 %) of the HIV physicians intended to initiate preventive treatment. |  |  | 🗸 |  | 🗸 |  |  |  | 🗸 |  |  |  |
|  | One in five (20%) of the HIV physicians would not initiate preventive treatment in any of the treatment scenarios. |  |  | 🗸 |  | 🗸 |  |  |  | 🗸 |  |  |  |
|  | Barrier 1: Physician factors  12 of the 24 (50 %) respondents gave an outspoken negative answer on the question whether the guideline influenced their patient care (self-efficacy). |  |  | 🗸 |  |  | 🗸 |  |  | 🗸 |  |  |  |
|  | Barrier 2: Guideline factors  Only 21 % (5/24) of the physicians reported the guideline recommendations to correspond to their daily practice.  46 % (11/24) of the respondents reported adequate involvement with the formulation of the guideline (in person or through the professional body)  8 % (2/24) denied any involvement at all. |  |  | 🗸 |  |  | 🗸 |  | 🗸 |  |  |  |  |
|  | Barrier 3: Patient factors  The a priori risk for LTBI was reported to drive the decisions regarding intended screening and treatment for LTBI, according to 83 % (20/24) of the respondents, while the clinical presentation of patients was a decisive factor for 58 % (14/24) of the respondents. Although the respondents reported that patients almost never refuse screening for LTBI, they do sometimes refuse preventive treatment. |  |  | 🗸 | 🗸 |  |  |  |  | 🗸 |  |  |  |
|  | Barrier 4: Environmental factors  There are no incentives for adherence to the guideline. |  |  | 🗸 |  | 🗸 |  |  |  |  |  | 🗸 |  |
| Gao 2015 [42] | What is the main symptom that indicates latent TB infection?  **No symptoms (correct): 181 (19.8%)**  Incorrect (cough ≥3 weeks, cough with blood, fever, night sweats, weight loss, diarrhea, and do not know): 717 (78.6%) | 🗸 |  |  | 🗸 |  |  |  | 🗸 |  |  |  |  |
|  | Can latent TB infection be spread from person to person?  **No (correct): 173 (19.0%)**  Incorrect: 713 (78.2%) | 🗸 |  |  | 🗸 |  |  |  | 🗸 |  |  |  |  |
|  | Latent TB infection can be treated with?  **Prescribed TB medicine (correct): 583 (63.9%)**  Incorrect (general antibiotics, there is no treatment, herbal remedies, bed rest, acupuncture, and do not know): 316 (34.6%) | 🗸 |  |  | 🗸 |  |  |  | 🗸 |  |  |  |  |
|  | The benefit of treating latent TB infection is?  **To prevent active TB disease (correct): 401 (44.0%)**  Incorrect: 500 (54.8%) | 🗸 |  |  | 🗸 |  |  |  | 🗸 |  |  |  |  |
|  | The treatment of latent TB infection lasts?  **3–6 months/>6 months (correct): 378 (41.4%)**  Incorrect: 517 (56.7%) | 🗸 |  |  | 🗸 |  |  |  | 🗸 |  |  |  |  |
|  | Do you think BCG vaccine (a vaccine for TB) completely protects you from TB for your whole life?  **No (correct): 495 (54.3%)**  Incorrect: 399 (43.8%) | 🗸 |  |  | 🗸 |  |  |  | 🗸 |  |  |  |  |
| Gupta 2011 [9] | 38/44 of clinicians screened patients for LTBI prior to the use of TNF inhibitors, although this was independent of the particular method used to screen for LTBI (which included merely asking about risk factors for LTBI). |  |  | 🗸 |  | 🗸 |  |  |  | 🗸 |  |  |  |
|  | When screening these patients,  34/41 clinicians requested a chest X-ray;  6/41 clinicians performed a chest X-ray if risk factors for LTBI were present.  More used the QuantiFERON-TB Gold (27/39; plus 6/39 who tested those patients with risk factors only) than the Mantoux test (11/27 and 5/27 respectively). |  |  | 🗸 |  |  | 🗸 | 🗸 |  |  |  |  |  |
| Gutsfeld 2014 [10] | 291 (81.1%) physicians agreed testing persons at risk with TST/IGRA and treating individuals with a positive test result is an efficient method of prevention. |  | 🗸 |  |  | 🗸 |  |  |  | 🗸 |  |  |  |
|  | Experience of using LTBI screening tools:  TST: 57.1%  IGRA QuantiFeron Gold in tube: 69.4%  IGRA T-Spot.TB: 27.5% |  |  | 🗸 |  | 🗸 |  | 🗸 |  |  |  |  |  |
|  | Future intention / preference of LTBI screening tools:  TST: 34.1%  IGRA QuantiFeron Gold in tube: 67.7%  IGRA T-Spot.TB: 32.2% |  |  | 🗸 |  |  | 🗸 | 🗸 |  |  |  |  |  |
|  | TB preventive treatment regimens choice:  The preferred preventive treatment is isoniazid for 9 months (n = 194/362; 54.4%), followed by isoniazid for 6 months (n= 85; 23.5%) and treatment with the combination of daily treatment with isoniazid and rifampicin for 3 months (n =46; 12.7%).  15 physicians (4.1%) favored daily  treatment with isoniazid monotherapy for 12 months and 5 physicians (1.4%) daily treatment with rifampicin monotherapy for 4 months. |  |  | 🗸 |  |  | 🗸 | 🗸 |  |  |  |  |  |
|  | 208 (58.4%) physicians avoid to administer preventive treatment for the risks of side effects. |  | 🗸 |  |  | 🗸 |  |  |  |  |  | 🗸 |  |
|  | 144 (40.9%) physicians have no insight into the efficacy of preventive treatment. |  | 🗸 |  |  | 🗸 |  |  | 🗸 |  |  |  |  |
|  | Patient factor:  247 (69%) physicians agreed patients were hesitate to enter preventive treatment for the fear of side effects. |  | 🗸 |  | 🗸 |  |  |  |  |  |  | 🗸 |  |
|  | Suggestion to improve TB prevention:  173 (57.5%) physicians favored improvements in the diagnostics of LTBI |  |  | 🗸 |  |  | 🗸 |  |  |  |  | 🗸 |  |
|  | Suggestion to improve TB prevention:  129 (41%) physicians favored a stronger efficacy of preventive treatment regimens |  |  | 🗸 |  |  | 🗸 |  |  |  |  | 🗸 |  |
|  | Suggestion to improve TB prevention:  117 (39%) physicians favoured improvements to shorten the duration of preventive chemotherapy |  |  | 🗸 |  | 🗸 |  |  |  | 🗸 |  |  |  |
| Hill 2010 [43] | Participants (TST positive youth) reported a general lack of knowledge of LTBI and TB at the initial interview. | 🗸 |  |  | 🗸 |  |  |  | 🗸 |  |  |  |  |
|  | Participants (TST positive youth):  15.2 % did not know that LTBI treatment reduces the risk of TB. | 🗸 |  |  | 🗸 |  |  |  | 🗸 |  |  |  |  |
|  | Patient factor:  Did not know that LTBI treatment reduces risk of TB: 15.1% | 🗸 |  |  | 🗸 |  |  |  | 🗸 |  |  |  |  |
|  | Parental factor:  The baseline questionnaire revealed a lack of parental knowledge regarding LTBI and LTBI treatment. | 🗸 |  |  | 🗸 |  |  |  | 🗸 |  |  |  |  |
|  | Parental factor:  20% of the participating parents did not know that LTBI could be detected by a skin test. | 🗸 |  |  | 🗸 |  |  |  | 🗸 |  |  |  |  |
|  | Parental factor:  13% did not know that completing LTBI treatment greatly reduces risk of TB. | 🗸 |  |  | 🗸 |  |  |  | 🗸 |  |  |  |  |
|  | Parental factor:  26% were ‘not worried when they found out their child had a positive TST.’ |  | 🗸 |  | 🗸 |  |  |  |  |  | 🗸 |  |  |
|  | Parental factor:  40% of parents did not think that positive TST reflected being ‘infected with TB bacteria at some time in the past.’ | 🗸 |  |  | 🗸 |  |  |  | 🗸 |  |  |  |  |
|  | Parental factor:  14% of parents thought positive TST was from ‘receiving BCG vaccine as a child.’ | 🗸 |  |  | 🗸 |  |  |  | 🗸 |  |  |  |  |
|  | Parental factor:  19% of parents thought positive TST was attributed it to ‘scratching the site.’ | 🗸 |  |  | 🗸 |  |  |  | 🗸 |  |  |  |  |
|  | Parental factor:  Difficulties in scheduling appointments for the CXR and isoniazid due to:  (i) Parents’ work conflicts;  (ii) Lack of transportation  Lack of attendance to medical visits due to costs to see a provider, even the modest charges of community health centers.  One parent’s concerns about potential side effects in their child prompted cessation of LTBI treatment and dropping out of the study. |  | 🗸 |  | 🗸 |  |  |  |  |  |  | 🗸 |  |
|  | Funding factor:  Due to shrinking public health funds in general as well as those for TB control, CDC’s priorities narrowed to active tuberculosis treatment and contact tracing and care. The County is now only able to provide limited direct LTBI care, and does no subcontracting.  Per parental reports, even the modest charges of the participating CHCs for LTBI treatment were prohibitive for some participants.  Similarly, providing care at discounted rates that families could afford was prohibitive for many of the community clinics to afford. |  |  | 🗸 |  |  | 🗸 |  |  |  |  | 🗸 |  |
|  | Provider factor (TST positive youth):  Participants provided feedback on their contacts with physicians when attempting to get started on isoniazid,  reflecting **misinformation** and mistreatment, contributed to refusal to enroll in the study by at least 16 potential participants.  This included inappropriate dosing, not scheduling monthly visits, inappropriate length of treatment regimens, and inappropriate staff and/or methods of obtaining medical histories of possible side effects of isoniazid;  Some participants were told: ‘‘Adolescents don’t need treatment for LTBI’’, ‘‘Only 6 months of treatment is needed,’’ ‘‘Isoniazid is too toxic to use in adolescents.’’;  Several participants heard from provider: ‘‘The positive TST in Mexicans is from BCG, and your child doesn’t need treatment’’ and ‘‘Don’t take medicine, just get a CXR every year.’’ | 🗸 |  |  |  | 🗸 |  |  | 🗸 |  |  |  |  |
|  | Provider factor:  Additional problems were reported by parents and participants regarding obtaining refills. In some cases, families were told that medication was ready, but when they arrived at the facility to pick it up, there was no prescription.  Patients attending facilities using a mail system, where medication refills of isoniazid were mailed to the patient’s home, reported never receiving the refills.  Suggestion: Physicians need ongoing training to understand and implement policies for screening and appropriate treatment. |  |  | 🗸 |  | 🗸 |  | 🗸 |  |  |  |  |  |
|  | Provider factor:  Patients reported sometimes being asked about side effects by a nurse, a medical assistant, a receptionist, or not at all. |  |  | 🗸 |  |  | 🗸 | 🗸 |  |  |  |  |  |
| Hirsch-Moverman 2006 [11] | LTBI treatment protects against TB  Yes: 113 (69.8%) | 🗸 |  |  |  | 🗸 |  |  | 🗸 |  |  |  |  |
|  | Risks of LTBI treatment higher than benefits  Yes: 20 (12.3%) |  | 🗸 |  |  | 🗸 |  |  |  | 🗸 |  |  |  |
|  | LTBI treatment should be mandatory for TST positive new immigrants  Yes: 90 (55.6%) |  | 🗸 |  |  | 🗸 |  |  |  | 🗸 |  |  |  |
|  | For respondents with BCG and TST positive, your TST positive is caused by BCG  Yes: 15 (19.5%), suggesting misperception | 🗸 |  |  |  | 🗸 |  |  | 🗸 |  |  |  |  |
|  | For respondents with BCG and TST positive, BCG protects you against TB  Yes: 23 (29.9%), suggesting misperception | 🗸 |  |  |  | 🗸 |  |  | 🗸 |  |  |  |  |
| Hirsch-Moverman 2013 [31] | Importance in testing preferences (Moderate to high importance):  Accuracy of results: 383 (94.3%) |  | 🗸 |  |  | 🗸 |  |  |  | 🗸 |  |  |  |
|  | Importance in testing preferences (Moderate to high importance):  Ability to understand what results mean: 372 (91.6%) |  | 🗸 |  |  | 🗸 |  |  |  | 🗸 |  |  |  |
|  | Importance in testing preferences (Moderate to high importance):  Effect on ability to work: 331 (81.5%) |  | 🗸 |  |  | 🗸 |  |  |  | 🗸 |  |  |  |
|  | Importance in testing preferences (Moderate to high importance):  Which test healthcare provider recommends: 314 (77.3%) |  | 🗸 |  |  | 🗸 |  |  |  |  |  | 🗸 |  |
|  | Importance in testing preferences (Moderate to high importance):  Ability to understand how test works: 314 (77.3%) |  | 🗸 |  |  | 🗸 |  |  |  | 🗸 |  |  |  |
|  | Importance in testing preferences (Moderate to high importance):  Side effects from test: 296 (72.9%) |  | 🗸 |  |  | 🗸 |  |  |  |  |  | 🗸 |  |
|  | Importance in testing preferences (Moderate to high importance):  Cost of test: 268 (66.0%) |  | 🗸 |  |  | 🗸 |  |  |  |  |  | 🗸 |  |
|  | Importance in testing preferences (Moderate to high importance):  Convenience of test: 265 (65.3%) |  | 🗸 |  |  | 🗸 |  |  |  |  |  | 🗸 |  |
|  | Importance in testing preferences (Moderate to high importance):  Amount of time test in use: 237 (58.4%) |  | 🗸 |  |  | 🗸 |  |  |  |  |  | 🗸 |  |
|  | Importance in testing preferences (Moderate to high importance):  Pain of test: 197 (48.5%) |  | 🗸 |  |  | 🗸 |  |  |  |  |  | 🗸 |  |
|  | Most of the participants (305/406; 75.2%) had not heard about IGRAs before joining the study, 77 (18.9%) had heard some information, and 22 (5.4%) had heard a lot. | 🗸 |  |  |  | 🗸 |  |  | 🗸 |  |  |  |  |
|  | After briefing, 203 (50.0%) participants  preferred an IGRA to the TST (87/406 or 21.4%); an additional 116 (28.5%) had no test preference or did not answer the question. |  | 🗸 |  |  | 🗸 |  |  |  | 🗸 |  |  |  |
|  | IGRA preference:  Convenience (47.8%),  Perceived accuracy (34.0%),  Combination of convenience and perceived accuracy (11.3%). |  | 🗸 |  |  | 🗸 |  |  |  | 🗸 |  |  |  |
|  | Preferring the TST:  Familiarity with the test (27.6%),  Convenience (24.1%),  Perception of it being less invasive or painful (23.0%). |  | 🗸 |  |  | 🗸 |  |  |  | 🗸 |  |  |  |
|  | Belief in a positive result of: TST: 69.2%  IGRA: 75.9% |  | 🗸 |  |  | 🗸 |  |  |  | 🗸 |  |  |  |
|  | Willingness to accept LTBI treatment if positive:  In TST: 79.3%  In IGRA 78.8% |  | 🗸 |  |  | 🗸 |  |  |  | 🗸 |  |  |  |
|  | HCWs expressed more confidence in IGRA results as compared to TST results.  TST positive + IGRA was negative: 53.4% of participants would believe an IGRA result; 14.5% would believe the TST result, with about a third (31.5%) being not sure.  TST negative + IGRA positive, 54.4% of participants would believe in an IGRA result and 9.9% would believe in the TST (9.9%) result. |  | 🗸 |  |  | 🗸 |  |  |  | 🗸 |  |  |  |
| Howley 2015 [49] | If people have latent TB infection, that is, sleeping TB germs, can they give TB to other people?  No: 137 (28.7%) | 🗸 |  |  | 🗸 |  |  |  | 🗸 |  |  |  |  |
|  | Can a person have TB germs in their body and not feel sick?  Yes: 388 (81.3%) | 🗸 |  |  | 🗸 |  |  |  | 🗸 |  |  |  |  |
|  | Are people with HIV more likely to develop TB than people who do not have HIV?  Yes: 258 (54.1%) | 🗸 |  |  | 🗸 |  |  |  | 🗸 |  |  |  |  |
|  | Does a positive TB skin test always mean that a person has TB disease?  No: 269 (56.4%) | 🗸 |  |  | 🗸 |  |  |  | 🗸 |  |  |  |  |
|  | Does a positive TB skin test usually mean that a person has TB germs in their body?  Yes: 361 (75.7%) | 🗸 |  |  | 🗸 |  |  |  | 🗸 |  |  |  |  |
|  | Are there medicines to prevent a person from getting TB?  Yes: 244 (51.2%) | 🗸 |  |  | 🗸 |  |  |  | 🗸 |  |  |  |  |
|  | TB is as serious as other worries in your life: 3.2±1.1  (Scoring scale - 1: Strongly disagree; 4: Strongly agree) |  | 🗸 |  | 🗸 |  |  |  |  |  | 🗸 |  |  |
|  | TB medicines will cause health problems: 2.9±1.1  (Scoring scale - 1: Strongly disagree; 4: Strongly agree) |  | 🗸 |  | 🗸 |  |  |  |  | 🗸 |  |  |  |
|  | You care what people close to you think of your TB treatment: 2.7±1.3  (Scoring scale - 1: Strongly disagree; 4: Strongly agree) |  | 🗸 |  | 🗸 |  |  |  |  |  |  |  | 🗸 |
|  | Having TB will greatly affect your life: 2.4±1.3  (Scoring scale - 1: Strongly disagree; 4: Strongly agree) |  | 🗸 |  | 🗸 |  |  |  |  |  | 🗸 |  |  |
|  | You feel ashamed to have TB: 1.9±1.1  (Scoring scale - 1: Strongly disagree; 4: Strongly agree) |  | 🗸 |  | 🗸 |  |  |  |  |  | 🗸 |  |  |
| Jackson 2007 [32] | 9. Generally, what percentage of people in the U.S. who have LTBI and a normal immune system will go on to develop TB disease at some point in their lives?  A. 1%: 364 (24.6%)  **B. 10% (correct answer): 944 (63.8%)**  C. 50%: 140 (9.5%)  D. 90%: 29 (2.0%) | 🗸 |  |  |  | 🗸 |  |  | 🗸 |  |  |  |  |
|  | Which of the following is a contraindication to TB skin testing?  A. BCG vaccination: 519 (35.1%)  B. TB disease: 388 (26.2%)  C. Malnutrition: 44 (3.0%)  **D. None of the above (correct answer): 526 (35.5%)**  Misperception on BCG vaccination | 🗸 |  |  |  | 🗸 |  |  | 🗸 |  |  |  |  |
|  | Patient care experiences:  >6 patients: 113  4 to 6 patients: 102  1 to 3 patients: 471  None: 645 |  |  | 🗸 |  | 🗸 |  | 🗸 |  |  |  |  |  |
| Kane 2013 [44] | 85.6% of participants believed that there was a chance that this (LTBI) could wake up and make them sick. | 🗸 |  |  | 🗸 |  |  |  | 🗸 |  |  |  |  |
|  | 90.4% of participants believed that the LTBI treatment would kill the LTBI in their lungs. | 🗸 |  |  | 🗸 |  |  |  | 🗸 |  |  |  |  |
|  | Provider factor:  **The amount of time spent at the clinic was too long**  Agree: 62.6%  Disagree: 37.4%  **The TB clinic staff listen to me and care about my health**  Agree: 3.6%  Disagree: 96.4% |  | 🗸 |  | 🗸 |  |  |  |  |  |  | 🗸 |  |
|  | Patient factor:  **Monthly clinic visits are difficult for me**  Agree: 62.6%  Disagree: 37.4%  **Sometimes I forget about clinic appointments**  Agree: 68.1%  Disagree: 31.9%  **I dislike having blood tests at clinic**  Agree: 63.6%  Disagree: 36.4%  **It is difficult to take time off work or college**  Agree: 47.0%  Disagree: 53.0%  **Getting to and fro hospital is expensive**  Agree: 60.0%  Disagree: 40.0% |  | 🗸 |  | 🗸 |  |  |  |  |  |  | 🗸 |  |
|  | It is important to take the TB medication everyday until the doctor tells me to stop  Agree: 8.1%  Disagree: 91.9% |  | 🗸 |  | 🗸 |  |  |  |  | 🗸 |  |  |  |
|  | I am worried about the side effects caused by TB medication  Agree: 17.0%  Disagree: 83.0% |  | 🗸 |  | 🗸 |  |  |  |  |  |  | 🗸 |  |
|  | I am happy to take TB medication on a daily basis for at least 6 months  Agree: 18.8%  Disagree: 81.2% |  | 🗸 |  | 🗸 |  |  |  |  |  |  | 🗸 |  |
| Karakousis 2007 [12] | The median (knowledge) score for all medical residents in the category of latent tuberculosis infection was 40.7%. | 🗸 |  |  |  | 🗸 |  |  | 🗸 |  |  |  |  |
|  | Interpretation of tuberculin skin test results posed particular difficulty for the participating medical residents. | 🗸 |  |  |  | 🗸 |  |  | 🗸 |  |  |  |  |
|  | When presented with a clinical vignette describing a foreign-born, BCG-vaccinated individual, whose tuberculin skin test is positive (Question #1), fewer than half of residents would treat with the appropriate regimen of isoniazid for 9 months (47%; n = 62).  Among those responding incorrectly to the above question, 71% believed that the positive tuberculin skin test was attributable to prior BCG vaccination, and recommended no specific therapy for latent tuberculosis infection. | 🗸 |  |  |  | 🗸 |  |  | 🗸 |  |  |  |  |
|  | 85 (66%) residents correctly recognized that a tuberculin skin test inducing a 5-mm reaction is not considered positive for health care workers (Question #5). | 🗸 |  |  |  | 🗸 |  |  | 🗸 |  |  |  |  |
|  | 64 (48.9%) residents believed incorrectly that the lifetime risk of developing active disease in non-HIV infected persons with latent tuberculosis infection was below 2 percent (Question #6). | 🗸 |  |  |  | 🗸 |  |  | 🗸 |  |  |  |  |
|  | Many residents (39%, n = 51) incorrectly thought that the annual risk of developing active disease in HIV-infected patients with latent tuberculosis infection was less than or equal to 1 percent (Question #7). | 🗸 |  |  |  | 🗸 |  |  | 🗸 |  |  |  |  |
|  | Only 41% of residents (n = 53) identified the combination of rifampin and pyrazinamide as an unacceptable regimen, and half of residents (n = 67) were aware of the acceptability of a 4-month regimen of rifampin for the treatment of latent tuberculosis infection (Question #4). | 🗸 |  |  |  | 🗸 |  |  | 🗸 |  |  |  |  |
|  | Only 15% (n = 108) were aware of the proper use of pyridoxine supplementation (Question #10), with three quarters of incorrect responders choosing not to recommend pyridoxine supplementation during pregnancy. | 🗸 |  |  |  | 🗸 |  |  | 🗸 |  |  |  |  |
|  | Many medical residents lack adequate knowledge of recommended guidelines for the management of TB.  Suggestion: Education of medical residents on guidelines for detection and early management of TB may be important for future improvements in national TB control strategies. |  |  | 🗸 |  |  | 🗸 |  | 🗸 |  |  |  |  |
| Lazar 2010 [13] | 156 (62.2%) reported being aware of the testing policies of the school district in which they practice;  161 (62.4%) were aware of new guidelines published;  146 (56.6%) read the new guidelines |  |  | 🗸 |  |  | 🗸 |  | 🗸 |  |  |  |  |
|  | Providers reported that 39% assess for LTBI during routine health assessments, 40% assess for LTBI during required school physical examinations, and 39% assess children for LTBI at every well child visit. |  |  | 🗸 |  |  | 🗸 | 🗸 |  |  |  |  |  |
|  | Most providers (76%) felt that the school district’s policy for TB testing is appropriate because they serve a low-risk population, 20% felt that the school district’s policy was appropriate because they serve a high-risk population. |  |  | 🗸 |  | 🗸 |  | 🗸 |  |  |  |  |  |
| Li 2018 [45] | Of the 560 student contacts, 487 (87.0%) agreed to receive chemo-prophylaxis if diagnosed with LTBI. |  | 🗸 |  | 🗸 |  |  |  |  | 🗸 |  |  |  |
|  | Willing to receive LTBI prophylaxis, because:  Preventing active TB: 327 (67.1%) | 🗸 |  |  | 🗸 |  |  |  | 🗸 |  |  |  |  |
|  | Willing to receive LTBI prophylaxis, because:  Concerns about spreading infection if TB reactivation: 241 (49.5%) | 🗸 |  |  | 🗸 |  |  |  | 🗸 |  |  |  |  |
|  | Willing to receive LTBI prophylaxis, because:  Consistent contact with TB patients: 241 (49.5%) | 🗸 |  |  | 🗸 |  |  |  | 🗸 |  |  |  |  |
|  | Willing to receive LTBI prophylaxis, because:  Doctors’ advice: 125 (25.7%) |  | 🗸 |  | 🗸 |  |  |  |  |  |  | 🗸 |  |
|  | Rejected LTBI prophylaxis because:  Not at risk of TB: 28 (38.4%) |  | 🗸 |  | 🗸 |  |  |  |  | 🗸 |  |  |  |
|  | Rejected LTBI prophylaxis because:  Unobservable effects of LTBI prophylaxis: 17 (23.3%) |  | 🗸 |  | 🗸 |  |  |  |  | 🗸 |  |  |  |
|  | Rejected LTBI prophylaxis because:  Prolonged duration of treatment: 16 (21.9%) |  | 🗸 |  | 🗸 |  |  |  |  |  |  | 🗸 |  |
|  | Rejected LTBI prophylaxis because:  Concerns about the cost of prophylaxis: 10 (13.7%) |  | 🗸 |  | 🗸 |  |  |  |  |  |  | 🗸 |  |
|  | Rejected LTBI prophylaxis because:  Concerns about the side effects: 9 (12.3%) |  | 🗸 |  | 🗸 |  |  |  |  |  |  | 🗸 |  |
|  | Rejected LTBI prophylaxis because:  Concerns about stress of treatment: 8 (11.0%) |  | 🗸 |  | 🗸 |  |  |  |  | 🗸 |  |  |  |
| Mirtskhulava 2015 [23] | The HCW overall average knowledge score was 61%. | 🗸 |  |  |  | 🗸 |  |  | 🗸 |  |  |  |  |
|  | The majority of HCWs (85%) knew the preferred regimen for LTBI treatment, but fewer (66%) knew the justification for latent TB therapy. | 🗸 |  |  |  | 🗸 |  |  | 🗸 |  |  |  |  |
|  | 70% of HCWs knew epidemiological, clinical, and laboratory characteristics of LTBI. | 🗸 |  |  |  | 🗸 |  |  | 🗸 |  |  |  |  |
|  | Only 43% of HCWs knew the risk of LTBI progression to TB disease. | 🗸 |  |  |  | 🗸 |  |  | 🗸 |  |  |  |  |
|  | Only 30% were able to correctly identify high-risk groups for LTBI progression to TB disease. | 🗸 |  |  |  | 🗸 |  |  | 🗸 |  |  |  |  |
|  | It is important to test individuals with  compromised immune systems for latent TB infection.  Agree: 195 (81.3%) |  | 🗸 |  |  | 🗸 |  |  |  | 🗸 |  |  |  |
|  | It is important for Georgian HCWs to be tested for latent TB infection.  Agree: 206  Neutral: 23  Disagree: 11 |  | 🗸 |  |  | 🗸 |  |  |  | 🗸 |  |  |  |
|  | It is important to test contacts of patients with TB (family, friends) for latent TB infection.  Agree: 216  Neutral: 15  Disagree: 9 |  | 🗸 |  |  | 🗸 |  |  |  | 🗸 |  |  |  |
|  | It is important to test children who have been exposed to TB for latent TB infection.  Agree: 221  Neutral: 16  Disagree: 3 |  | 🗸 |  |  | 🗸 |  |  |  | 🗸 |  |  |  |
|  | Worry about acquiring LTBI:  Agree: 132 (55.0%) |  | 🗸 |  |  | 🗸 |  |  |  |  | 🗸 |  |  |
|  | Latent TB infection is very serious.  Agree: 117 (48.8%) |  | 🗸 |  |  | 🗸 |  |  |  |  | 🗸 |  |  |
|  | LTBI screening: Would you be willing to be tested each year for latent TB infection?  Yes: 125 (52.1%) |  | 🗸 |  |  | 🗸 |  |  |  | 🗸 |  |  |  |
|  | LTBI treatment: If I test positive for latent TB infection, I should be treated.  Agree: 116 (48.3%) |  | 🗸 |  |  | 🗸 |  |  |  | 🗸 |  |  |  |
|  | Perceived barrier:  If I tested positive for LTBI, I should not be treated because I will be exposed again in the future.  Agree: 103 (42.9%)  Neutral: 50 (20.8%)  Disagree: 87 (36.3%) |  | 🗸 |  |  | 🗸 |  |  |  | 🗸 |  |  |  |
|  | Perceived barrier:  I tested positive for LTBI, I should not be treated because probably I have drug-resistant TB strains.  Agree: 66 (27.5%)  Neutral: 58 (24.2%)  Disagree: 116 (48.3%) |  | 🗸 |  |  | 🗸 |  |  |  | 🗸 |  |  |  |
|  | Perceived barrier:  Risks of treating LTBI outweigh benefits to treating LTBI.  Agree: 105 (43.8%)  Neutral: 84 (35.0%)  Disagree: 51 (21.3%) |  | 🗸 |  |  | 🗸 |  |  |  | 🗸 |  |  |  |
| Montagna 2014 [34] | TST is aimed at detecting asymptomatic *M. tuberculosis* infection.  88% declared that TST is helpful in diagnosing latent TB infection. | 🗸 |  |  |  | 🗸 |  |  | 🗸 |  |  |  |  |
|  | TB vaccine (BCG) is currently available, but it is not 100% effective.  Yes: 66.3% | 🗸 |  |  |  | 🗸 |  |  | 🗸 |  |  |  |  |
|  | 66.9% of the enrolled students reported to have experienced— in the past—a screening test for the diagnosis of LTBI through the Mantoux test (93%), the Tine test (2.9%) or both tests (1.8%). |  |  | 🗸 |  | 🗸 |  | 🗸 |  |  |  |  |  |
|  | It is crucial to plan and implement strategies and policies to improve the knowledge of health care team members towards TB, including the capacity to integrate knowledge and good practices |  |  | 🗸 |  |  | 🗸 |  |  | 🗸 |  |  |  |
| Montagna 2018 [33] | 84.4% of the study population (95% CI = 83.3-85.3) was aware of the existence of the tuberculin skin test, 74.4% (95% CI = 73.2-75.6) knew what is the first-level screening test for latent tuberculosis (i.e. Mantoux test) and only 22.5% (95% CI = 21.4-23.6) knew how to proceed after a positive tuberculin skin test result, i.e. IGRA test. |  |  | 🗸 |  | 🗸 |  | 🗸 |  |  |  |  |  |
|  | More than a third (39%) of students attending University were unaware of a mandatory TB screening policy. |  |  | 🗸 |  |  | 🗸 |  | 🗸 |  |  |  |  |
| Moolphate 2013 [24] | Barrier to IPT programme: unclear direction of national policy (60%) |  |  | 🗸 |  |  | 🗸 |  | 🗸 |  |  |  |  |
|  | Barrier to IPT programme: fear of emerging Isoniazid resistant tuberculosis (52%) |  | 🗸 |  |  | 🗸 |  |  |  | 🗸 |  |  |  |
|  | Barrier to IPT programme: fear of poor adherence (30%) |  | 🗸 |  |  | 🗸 |  |  |  |  |  | 🗸 |  |
|  | Barrier to IPT programme: fear of toxicity of Isoniazid (18%) |  | 🗸 |  |  | 🗸 |  |  |  |  |  | 🗸 |  |
|  | IPT programme was supported:  knowledge that IPT can prevent TB (63%) | 🗸 |  |  |  | 🗸 |  |  | 🗸 |  |  |  |  |
|  | IPT programme was supported:  the following of national guideline (34%) |  |  | 🗸 |  |  | 🗸 |  | 🗸 |  |  |  |  |
|  | IPT programme was supported:  concern for TB prevention even after the expansion of access to antiretroviral therapy (ART) (32%). |  |  | 🗸 |  | 🗸 |  |  | 🗸 |  |  |  |  |
|  | No IPT due to lack of training for HCW resulting in a lack of confidence in  providing IPT: 51% agreed |  |  | 🗸 |  | 🗸 |  | 🗸 |  |  |  |  |  |
|  | Have you ever seen the WHO IPT implementation guidelines?  Yes, in practice (17; 8.6%);  Yes, not in practice: Yes (27; 13.6%) |  |  | 🗸 |  | 🗸 |  |  | 🗸 |  |  |  |  |
| Narayanan 2019 [47] | Belief that BCG vaccine will protect from TB for whole life:  **No: 74 (21.6%)**  Yes: 123 (35.9%)  Do not know: 146 (42.6%) | 🗸 |  |  | 🗸 |  |  |  | 🗸 |  |  |  |  |
|  | Willingness to get tested for TB for free  Yes: 222 (63.4%) |  | 🗸 |  | 🗸 |  |  |  |  | 🗸 |  |  |  |
|  | Belief that it is important to get tested for TB (among non-US born South Asians from high TB endemic countries): 281 (77.4%) |  | 🗸 |  | 🗸 |  |  |  |  | 🗸 |  |  |  |
| O’Donnell 2011 [46] | 148/230 (64%) participants stated a preference for TST (148/230; 64.3%) compared to QFT-G (81/230; 35.2%). |  | 🗸 |  | 🗸 |  |  |  |  | 🗸 |  |  |  |
|  | Participants would accept treatment for LTBI based on:  TST: 47/230 (20.4%)  QFT-G: 26/230 (11.3%)  Either QFT-G or TST: 157/230 (68.3%) |  | 🗸 |  | 🗸 |  |  |  |  | 🗸 |  |  |  |
|  | TB medicine could make me sick:  Agree: 46 (20.0%)  Neutral: 100 (43.5%)  Disagree: 84 (36.5%) |  | 🗸 |  | 🗸 |  |  |  |  | 🗸 |  |  |  |
| Pathak 2016 [25] | HCWs who knew the difference between active and latent TB?  Yes: 242/306 (79%) | 🗸 |  |  |  | 🗸 |  |  | 🗸 |  |  |  |  |
|  | Experience with LTBI screening:  258/295 (87%) HCWs had undergone TST and/or IGRA testing. Of those who underwent a TST and/or IGRA test, 116 (45%) indicated that they had a positive screening test result suggestive of LTBI. |  | 🗸 |  |  | 🗸 |  |  |  | 🗸 |  |  |  |
|  | LTBI treatment:  81% (198/244) of respondents were in favour of being offered preventive TB treatment if they had evidence of LTBI personally and 19% (46/244) preferred not be offered preventive TB treatment personally. |  | 🗸 |  |  | 🗸 |  |  |  | 🗸 |  |  |  |
|  | LTBI treatment:  Only 13% (14/106) of HCWs with evidence of LTBI indicated that they were offered preventive TB treatment; 40% (42/106) were not offered treatment, and 47% (50/106) felt that this question did not apply to them. |  | 🗸 |  |  | 🗸 |  |  |  | 🗸 |  |  |  |
|  | LTBI treatment acceptance:  64% of HCWs (9/14) accepted the offered preventive TB treatment. |  | 🗸 |  |  | 🗸 |  |  |  | 🗸 |  |  |  |
|  | Reasons of rejecting LTBI treatment:  They could monitor signs and symptoms of TB themselves (3/5) |  | 🗸 |  |  | 🗸 |  |  |  | 🗸 |  |  |  |
|  | Reasons of rejecting LTBI treatment:  They feared side effects (2/5) |  | 🗸 |  |  | 🗸 |  |  |  |  |  | 🗸 |  |
|  | Reasons of rejecting LTBI treatment:  Felt that the treatment was unnecessary (2/5) |  | 🗸 |  |  | 🗸 |  |  |  | 🗸 |  |  |  |
|  | Reasons of rejecting LTBI treatment:  They did not need treatment as they had been vaccinated (1/5). | 🗸 |  |  |  | 🗸 |  |  | 🗸 |  |  |  |  |
|  | Importance for HCWs to take LTBI treatment:  74% (164/223) supported the idea that hospital staff who had evidence of LTBI should receive preventive treatment while 26% (59/223) did not think that preventive TB treatment was indicated in this situation. |  | 🗸 |  |  | 🗸 |  |  |  | 🗸 |  |  |  |
| Quirós 2018 [14] | Which candidates for biological therapies undergo latent tuberculosis infection screening?  Correct:  Latent tuberculosis infection screening is performed in all patients who are candidates for biological therapies, but not always before starting treatment: 93.7% | 🗸 |  |  |  | 🗸 |  |  | 🗸 |  |  |  |  |
|  | What tests do you request for latent tuberculosis infection screening?  Correct:  Chest X-ray + TST + IGRA: 36.6%  Incorrect:  Chest X-ray + TST: 45.6%  Chest X-ray + IGRA: 11.4% |  |  | 🗸 |  |  | 🗸 | 🗸 |  |  |  |  |  |
|  | If the TST is negative, what do you do next?  Correct:  Request IGRA before starting treatment with biologics: 26.1%  Incorrect:  Repeat TST after 2weeks due to the booster effect: 57.6% |  |  | 🗸 |  |  | 🗸 | 🗸 |  |  |  |  |  |
|  | If the TST is positive, what do you do next?  Correct:  Administer chemoprophylaxis for at least 4 weeks before starting treatment with biologics, after ruling out active TB: 63.9%  Incorrect:  Administer chemoprophylaxis for only 2 weeks before starting biologics: 15.4% |  |  | 🗸 |  |  | 🗸 | 🗸 |  |  |  |  |  |
|  | If chemoprophylaxis is prescribed, what regimen do you use most often?  96%replied correctly; most respondents would prescribe either isoniazid (H) in monotherapy (83%) or combined H and rifampicin (R) in special cases.  The treatment duration proposed by most respondents (9 and 3 months, respectively) complied with the national consensus. | 🗸 |  |  |  | 🗸 |  |  | 🗸 |  |  |  |  |
|  | 20% of the respondents reported they would not start chemoprophylaxis in cases in whom it should be indicated, as these are specialists who have less experience in the management of TB. |  |  | 🗸 |  | 🗸 |  | 🗸 |  |  |  |  |  |
|  | IGRA testing is not fully integrated into routine clinical practice. |  |  | 🗸 |  |  | 🗸 |  |  | 🗸 |  |  |  |
| Ramos 2018 [26] | 59 (58.4%) received formal training in TB; 58 of them had received training in TB prevention and LTBI management.  Despite having received formal training, less than 50% of the participants answered 7 of the 16 questions satisfactorily. |  |  | 🗸 |  |  | 🗸 | 🗸 |  |  |  |  |  |
|  | Knowledge gaps included prevention of LTBI among contacts (51%), prevention of progression to disease after infection (32%), LTBI diagnosis (43%), indications for LTBI treatment (62%), minimum duration of isoniazid treatment (44%), isoniazid dose (84%), and management of adverse events (57%). | 🗸 |  |  |  | 🗸 |  |  | 🗸 |  |  |  |  |
|  | How can a person with LTBI be prevented from becoming ill?  Satisfactory answer: 68% | 🗸 |  |  |  | 🗸 |  |  | 🗸 |  |  |  |  |
|  | What tests are recommended for an asymptomatic contact?  Satisfactory answer: 57% | 🗸 |  |  |  | 🗸 |  |  | 🗸 |  |  |  |  |
|  | How do you determine that a TB contact has been infected (with LTBI)?  Satisfactory answer: 46% | 🗸 |  |  |  | 🗸 |  |  | 🗸 |  |  |  |  |
|  | According to the recommendations of the Brazilian National Tuberculosis Control Program, what household contacts should receive treatment for LTBI?  Satisfactory answer: 38% | 🗸 |  |  |  | 🗸 |  |  | 🗸 |  |  |  |  |
|  | LTBI treatment:  What is the minimum duration of LTBI treatment with isoniazid to prevent TB disease?  Satisfactory answer: 56%  What is the optimal duration of LTBI treatment with isoniazid to prevent TB disease?  Satisfactory answer: 7%  What is the recommended dose of isoniazid for the treatment of LTBI in children?  Satisfactory answer: 16%  What is the daily dose of isoniazid for the treatment of LTBI in adults?  Satisfactory answer: 7%  What are the most common side effects of isoniazid?  Satisfactory answer: 82% | 🗸 |  |  |  | 🗸 |  |  | 🗸 |  |  |  |  |
|  | Do you think it is important for a child who lives with a patient with active TB to be screened for LTBI?  Yes: 54% |  | 🗸 |  |  | 🗸 |  |  |  | 🗸 |  |  |  |
|  | Do you think it is important for an adult who lives with a patient with active TB to be screened for LTBI?  Yes: 51% |  | 🗸 |  |  | 🗸 |  |  |  | 🗸 |  |  |  |
| Salazar-Schicchi 2004 [15] | LTBI treatment protects against TB  Correct answer:  Yes: 45/76 (59.2%) | 🗸 |  |  |  | 🗸 |  |  | 🗸 |  |  |  |  |
|  | For respondents with BCG and TST, your TST is caused by BCG:  Yes (incorrect): 11/40 (27.5%)  No (correct): 29/40 (72.5%) | 🗸 |  |  |  | 🗸 |  |  | 🗸 |  |  |  |  |
|  | For respondents with BCG and TST, BCG protects you against TB  Yes: 20/40 (50.0%)  No (correct): 20/40 (50.0%) | 🗸 |  |  |  | 🗸 |  |  | 🗸 |  |  |  |  |
|  | BCG:  Does not protect against TB: 11 (14.3%)  Protect for short while: 42 (54.5%)  Protect for many years: 20 (26.0%)  Forever: 0 | 🗸 |  |  |  | 🗸 |  |  | 🗸 |  |  |  |  |
|  | Risks of LTBI treatment higher than benefits:  Disagreed (correct): 63/75 (84.0%) |  | 🗸 |  |  | 🗸 |  |  |  | 🗸 |  |  |  |
|  | LTBI treatment should be mandatory for  TST positive in new immigrants:  Agreed: 39/75 (52.0%) |  | 🗸 |  |  | 🗸 |  |  |  | 🗸 |  |  |  |
| Skinner 2013 [50] | Although 59% of the participants had been previously treated for TB, only 33% had ever heard of IPT prior to this episode. | 🗸 |  |  | 🗸 |  |  |  | 🗸 |  |  |  |  |
|  | 95% of the participants understood that IPT meant giving a child a pill every day for 6 months. | 🗸 |  |  | 🗸 |  |  |  | 🗸 |  |  |  |  |
|  | Almost all participants intended to give  their child IPT (97%). |  | 🗸 |  | 🗸 |  |  |  |  | 🗸 |  |  |  |
|  | 91% of the participants had a  positive attitude towards IPT. |  | 🗸 |  | 🗸 |  |  |  |  | 🗸 |  |  |  |
|  | IPT was perceived to reduce a child’s chance of becoming ‘ill with TB’ (84%) and/or ‘dying due to TB’ (86%). | 🗸 |  |  | 🗸 |  |  |  | 🗸 |  |  |  |  |
|  | Only 6% were concerned about medication side effects. |  | 🗸 |  | 🗸 |  |  |  |  |  |  | 🗸 |  |
|  | Participants recognised that children were more likely than adults to become  very sick (94%) and/or die (77%) from TB. Participants also recognised a child’s risk of ‘catching TB’ from adult visitors (89%), household members (97%) and adults sleeping in the same room (94%). | 🗸 |  |  | 🗸 |  |  |  | 🗸 |  |  |  |  |
|  | Participants were concerned that providing IPT to their child would result in stigma to the child (42%) or themselves (36%). |  | 🗸 |  | 🗸 |  |  |  |  |  |  |  | 🗸 |
| Smith 2012 [16] | More than 87% of the specialists reported recognizing TB reactivation as a risk associated with anti-TNF agents. | 🗸 |  |  |  | 🗸 |  |  | 🗸 |  |  |  |  |
|  | More than 70% prescribers followed TB screening guidelines prior to prescribing an anti-TNF agent. |  |  | 🗸 |  |  | 🗸 |  | 🗸 |  |  |  |  |
|  | 30 to 65% of prescribers re-tested patients for TB following initiation of anti-TNF treatment. |  |  | 🗸 |  |  | 🗸 | 🗸 |  |  |  |  |  |
|  | The average time prescribers to re-test ranged from 12 months to 27.3 months. |  |  | 🗸 |  |  | 🗸 | 🗸 |  |  |  |  |  |
| Spruijt 2020 [27] | Total clients who initiated LTBI treatment: 49/94 (52.1%)  Suboptimal LTBI treatment initiation |  | 🗸 |  |  | 🗸 |  |  |  | 🗸 |  | 🗸 |  |
|  | Rejected LTBI treatment because:  Objection against long duration of IPT / afraid of side-effects: 6 (13%) |  |  |  |  | 🗸 |  |  |  |  |  |  |  |
|  | Rejected LTBI treatment because:  No perceived advantages of LTBI treatment by the client: 16 (36%)  TB physicians argued that for immigrants with a short-intended stay and high risk for re-infection in the home country, the individual and public health benefits would not outweigh the risks of LTBI treatment. |  | 🗸 |  |  | 🗸 |  |  |  | 🗸 |  |  |  |
|  | Challenges during LTBI treatment:  Difficulties with follow-up appointments: 6 (12%) |  | 🗸 |  |  | 🗸 |  |  |  |  |  | 🗸 |  |
|  | Challenges during LTBI treatment:  Interruption of treatment: 3 (6%) |  | 🗸 |  |  | 🗸 |  |  |  |  |  | 🗸 |  |
|  | Challenges during LTBI treatment:    Difficulties with duration of LTBI treatment: 2 (4%) |  | 🗸 |  |  | 🗸 |  |  |  |  |  | 🗸 |  |
|  | Challenges during LTBI treatment:  No understanding of difference LTBI and active TB: 1 (2%) | 🗸 |  |  |  | 🗸 |  |  | 🗸 |  |  |  |  |
| Stout 2006 [17] | 46% physicians had not treated any young children with LTBI. |  |  | 🗸 |  |  | 🗸 | 🗸 |  |  |  |  |  |
|  | Most (83%) physicians routinely placed tuberculin skin tests (TSTs), and 26% reported placing >10 TSTs per month. |  |  | 🗸 |  |  | 🗸 | 🗸 |  |  |  |  |  |
|  | Experience:  How often a child with LTBI seen:  **Never: 69;**  **Rarely: 59;**  Sometimes: 18;  Often: 2  Place TST:  Nurse: 116  Physician: 14  Read TST:  Nurse: 102  Physician: 78 |  |  | 🗸 |  | 🗸 |  | 🗸 |  |  |  |  |  |
|  | Physicians were particularly confused about: 1) TST among bacille Calmette-Guérin (BCG) vaccinated children. | 🗸 |  |  |  | 🗸 |  | 🗸 |  |  |  |  |  |
|  | Physicians were particularly confused about: 2) treatment of young children  with recent exposure to an adult with infectious. | 🗸 |  |  |  | 🗸 |  | 🗸 |  |  |  |  |  |
|  | TST is contraindicated in children with BCG (wrong statement):  70% disagree (correct)  30% agree or no opinion (incorrect) | 🗸 |  |  |  | 🗸 |  | 🗸 |  |  |  |  |  |
| Trajman 2019 [29] | What is difference between active TB and LTBI?  Satisfactory answer: 50 (37%), details as below:  The person with latent TB has no symptoms (correct): 27 (20%)  The person with TB has symptoms such as cough, fever, weight loss: 43 (32%)  Does not know the difference: 65 (48%)  The person with TB has symptoms and contacts with LTBI do not: 29 (21%) | 🗸 |  |  |  | 🗸 |  | 🗸 |  |  |  |  |  |
|  | How can one affirm that the person is infected with the tuberculosis bacillus?  Satisfactory answer: 50 (37%), details as below:  Person with positive tuberculin skin test (“PPD”) or IGRA: 37 (27%)  Person with positive tuberculin skin test (PPD) or IGRA and normal chest X-ray: 8 (6%)  Asymptomatic person with positive tuberculin skin test (PPD) or IGRA and normal chest X-ray: 15 (11%) | 🗸 |  |  |  | 🗸 |  | 🗸 |  |  |  |  |  |
|  | How do you prevent a person - once infected - from becoming ill with TB?  Satisfactory answer: 65 (48%), details as below:  Sleeping in a bed different from that of the TB patient: 1 (1%)  Taking isoniazid or other treatment for latent infection (chemoprophylaxis): 37 (27%)  With medicines (does not know the name): 30 (22%) | 🗸 |  |  |  | 🗸 |  | 🗸 |  |  |  |  |  |
|  | Adults are afraid to come for investigation because of fear of stigma: 45 (33.3%) |  | 🗸 |  | 🗸 |  |  |  |  |  |  |  | 🗸 |
|  | Parents / guardians of children may not bring them to the investigation because of fear of stigma: 13 (10%) |  | 🗸 |  | 🗸 |  |  |  |  |  |  |  | 🗸 |
|  | Do you think it is important for a child who lives with a patient with active TB to be screened for latent TB?    Yes = Satisfactory: 129 (96%) |  | 🗸 |  |  | 🗸 |  |  |  | 🗸 |  |  |  |
|  | Do you think it is important for an adult living with an active TB patient to be screened for latent TB?  Yes = Satisfactory: 133 (99%) |  | 🗸 |  |  | 🗸 |  |  |  | 🗸 |  |  |  |
|  | Although perceived barriers to investigate adult (99%) and child (96%) contacts for LTBI:  11 (8%) do not perceive contact identification and referral as part of  their attributed work;  11 (8%) reported being too busy;  and 7 (5%) not having adequate training. |  |  | 🗸 |  |  | 🗸 | 🗸 |  |  |  |  |  |
| Tran 2017 [18] | The preferred screening method by most of the respondents was either tuberculin skin test (19%) or interferon γ release assay (32%) or both (49%). |  | 🗸 |  |  |  | 🗸 | 🗸 |  |  |  |  |  |
|  | Confirmation test:    CXR in all patients starting biologic agents: 291 (37.8%)    CXR in patients with positive LTBI test before starting biologic agents: 297 (38.7%)  CXR in patients with TB risk factor or never prescribe CXR: 173 (22.5%) |  |  | 🗸 |  |  | 🗸 | 🗸 |  |  |  |  |  |
|  | For LTBI treatment, 49% of the respondents would refer management to infectious disease specialist or the health department, 37% would initiate isoniazid for 9 or 12 months, and 14% would use isoniazid for 6 months. |  |  | 🗸 |  |  | 🗸 | 🗸 |  |  |  |  |  |
|  | Approximately 60% of respondents would initiate anti–tumor necrosis factor therapy after being on LTBI treatment for 1 month. |  |  | 🗸 |  |  | 🗸 | 🗸 |  |  |  |  |  |
|  | How often you provide screening for TB after initiation of anti–tumor necrosis:  Every 2 years: 116  Annually on all patients: 188  Annually on patients with risk factors: 182  Once prior to therapy: 281 |  |  | 🗸 |  |  | 🗸 | 🗸 |  |  |  |  |  |
| Vinnard 2012 [19] | What are the annual screening practices? (n = 100)  All employees receive annual screening: 34 (34%)  Only employees with direct patient contact receive annual screening: 41 (41%)  Only baseline screening for LTBI: 14 (14%)  Another approach: 11 (11%) |  |  | 🗸 |  |  | 🗸 | 🗸 |  |  |  |  |  |
|  | What LTBI screening approach is used? (n = 94)  TSTs exclusively: 24 (26%)  IGRAs exclusively: 7 (7%)  Another sequential testing approach: 37 (39%)  IGRAs only for HCWs with a history of BCG vaccination: 26 (28%) |  |  | 🗸 |  |  | 🗸 | 🗸 |  |  |  |  |  |
|  | Who is offered LTBI treatment? (n = 102)  Only employees with a documented test conversion: 55 (54%)  All employees with a positive test, regardless of baseline testing status: 47 (46%) |  |  | 🗸 |  |  | 🗸 | 🗸 |  |  |  |  |  |
|  | Is IGRA available onsite? (n = 102)  Yes: 46 (45%)  No: 56 (55%) |  |  | 🗸 |  |  | 🗸 |  |  |  |  | 🗸 |  |
|  | Providers that use a sequential testing approach (n = 37)  IGRA as a confirmatory test for all employees with positive TST: 26 (70%)  IGRA as a confirmatory test for selected employees with positive TST: 10 (27%)  IGRA as a confirmatory test for all employees with negative TST: 1 (3%) |  |  | 🗸 |  |  | 🗸 | 🗸 |  |  |  |  |  |
|  | The cost of the test was an important factor in the choice of LTBI testing strategy at my facility.  **TST**  Attitude score: 3.4±1.2  (Scoring scale - 1: Strongly disagree; 4: Strongly agree)  **IGRA**  Attitude score: 3.9±0.96  (Scoring scale - 1: Strongly disagree; 4: Strongly agree) |  |  | 🗸 |  |  | 🗸 |  |  |  |  | 🗸 |  |
|  | Participants had neutral attitudes regarding treatment of HCWs with a history of BCG and a positive TST and regarding treatment of HCWs with recent conversions and negative IGRAs. |  | 🗸 |  |  | 🗸 |  |  | 🗸 |  |  |  |  |
|  | Participants were strongly inclined to treat HCWs with recent TST conversions and positive IGRAs. |  | 🗸 |  |  | 🗸 |  |  | 🗸 |  |  |  |  |
| Walker 2018 [48] | There may be persisting confusion about the role of bacille Calmette-Guerin vaccination (BCG) as 21 of 68 responding students agreed and 47 disagreed with the statement ‘I have had BCG vaccination and so I do not need the test’. | 🗸 |  |  | 🗸 |  |  | 🗸 |  |  |  |  |  |
|  | I think testing only migrants is discrimination.  Yes: 7  No: 59  Did not answer: 33 |  | 🗸 |  | 🗸 |  |  |  |  |  |  |  | 🗸 |
|  | I did not understand what the test was for.  Yes: 8  No: 59  Did not answer: 32  Lack of explanation or inadequate prior knowledge | 🗸 |  |  | 🗸 |  |  | 🗸 |  |  |  |  |  |
|  | I am well and do not need to be tested.  Yes: 33  No: 29  Did not answer: 37 |  | 🗸 |  | 🗸 |  |  |  |  | 🗸 |  |  |  |
| Xerinda 2016 [20] | Most of the participants were aware of tuberculosis risk and that they screened patients for tuberculosis following guidelines.  94 used Portuguese guidelines;  34 used European guidelines;  12 used American guidelines  However, there was a lack of awareness of guideline updates; together with fragmentation of health system (lack of communication and coordination with TB outpatient centre). |  |  | 🗸 |  |  | 🗸 |  | 🗸 |  |  |  |  |
|  | Use of IGRA for screening: 74 (77.9%) |  |  | 🗸 |  |  | 🗸 | 🗸 |  |  |  |  |  |
|  | 50 (52.6%) of the respondents did annual screening in their patients. |  |  | 🗸 |  |  | 🗸 | 🗸 |  |  |  |  |  |
|  | About half of them had training through courses, scientific journals and medical representatives. |  |  | 🗸 |  | 🗸 |  |  | 🗸 |  |  |  |  |
| Yates 2015 [21] | Among 114 resident physicians without a personal history of tuberculosis, 87 (76%) reported that they would accept LTBI treatment for themselves if the diagnosis were based on a positive TST, and 103 (90%) would accept LTBI treatment for themselves if the diagnosis was based on a positive IGRA. |  | 🗸 |  |  | 🗸 |  |  |  | 🗸 |  |  |  |
| Zhou 2014 [35] | Reason for declining a TST (n=94):  Previous positive TST results: 70.2% (66/94) |  | 🗸 |  |  | 🗸 |  |  |  |  |  | 🗸 |  |
|  | Reason for declining a TST (n=94):  Concerns about positive skin reactions, such as blisters, necrosis, and lymphadenitis: 31.9% (30/94) |  | 🗸 |  |  | 🗸 |  |  |  |  |  | 🗸 |  |
|  | Reason for declining a TST (n=94):  Receiving a TST in the past 3 months and anxiety about the psychological burden: 16.0% (15/94) |  | 🗸 |  |  | 🗸 |  |  |  |  | 🗸 |  |  |

TB: tuberculosis; SD: standard deviation; BCG: Bacillus Calmette–Guérin; INH: isoniazid; LTBI: latent tuberculosis infection; TST: tuberculin skin test; IGRA: interferon gamma release assay; HCW: healthcare worker; GP: general practitioner; the UK: the United Kingdom; IPT: isoniazid preventive therapy; TNF: tumor necrosis factor; CXR: chest X-ray; PPD: purified protein derivative

# Appendix 9 Figure S1 Intervention to improve latent tuberculosis management at system, provider and general public level mapped on to the subcomponents of Capability, Opportunity, and Motivation-Behaviour (COM-B) model


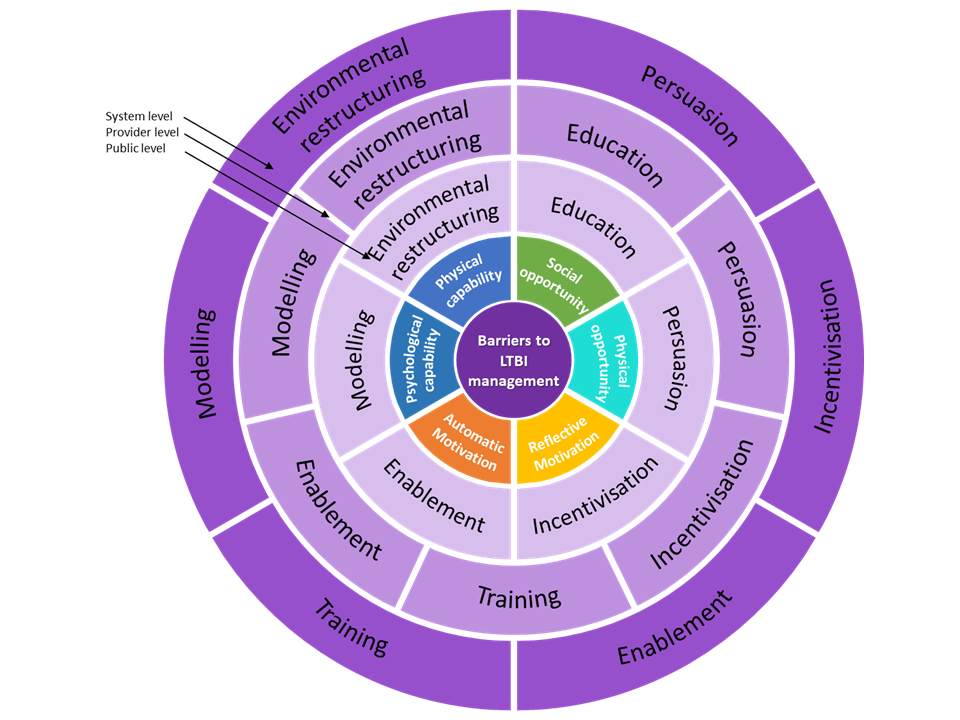


Figure S1 Intervention to improve latent tuberculosis management at system, provider and general public level mapped on to the subcomponents of Capability, Opportunity, and Motivation-Behaviour (COM-B) model

# References

1. Bennett DA. How can I deal with missing data in my study? *Aust N Z J Public Health* 2001: 25(5): 464-469.

2. Dong Y, Peng CY. Principled missing data methods for researchers. *Springerplus* 2013: 2(1): 222.

3. Hoy D, Brooks P, Woolf A, et al. Assessing risk of bias in prevalence studies: modification of an existing tool and evidence of interrater agreement. *J Clin Epidemiol* 2012: 65(9): 934-939.

4. Atchison C, Zenner D, Barnett L, Pareek M. Treating latent TB in primary care: a survey of enablers and barriers among UK General Practitioners. *BMC Infect Dis* 2015: 15.

5. Bhanot N, Haran M, Lodha A, Paul V, Goswami R, Chapnickt EK. Physicians' attitudes towards self-treatment of latent tuberculosis. *Int J Tuberc Lung Dis* 2012: 16(2): 169-171.

6. Cantini F, Lubrano E, Marchesoni A, et al. Latent tuberculosis infection detection and active tuberculosis prevention in patients receiving anti-TNF therapy: an Italian nationwide survey. *Int J Rheum Dis* 2016: 19(8): 799-805.

7. Cruz AT, Hersh AL, Starke JR, Beekmann SE, Polgreen PM, Banerjee R. Controversies in tuberculous infection among pediatric infectious disease specialists in North America. *Int J Tuberc Lung Dis* 2016: 20(11): 1463-1468.

8. Evenblij K, Verbon A, van Leth F. Intention of physicians to implement guidelines for screening and treatment of latent tuberculosis infection in HIV-infected patients in The Netherlands: a mixed-method design. *BMC Public Health* 2016: 16(1): 915.

9. Gupta A, Macrae FA, Gibson PR. Vaccination and screening for infections in patients with inflammatory bowel disease: a survey of Australian gastroenterologists. *Intern Med J* 2011: 41(6): 462-467.

10. Gutsfeld C, Olaru ID, Vollrath O, Lange C. Attitudes about Tuberculosis Prevention in the Elimination Phase: A Survey among Physicians in Germany. *PloS one* 2014: 9(11).

11. Hirsch-Moverman Y, Tsiouris S, Salazar-Schicchi J, Colson PW, Muttana H, El-Sadr W. Physician attitudes regarding latent tuberculosis infection: international vs. US medical graduates. *Int J Tuberc Lung Dis* 2006: 10(10): 1178-1180.

12. Karakousis PC, Sifakis FG, De Oca RM, et al. U. S. medical resident familiarity with national tuberculosis guidelines. *BMC Infect Dis* 2007: 7.

13. Lazar CM, Sosa L, Lobato MN. Practices and policies of providers testing school-aged children for tuberculosis, Connecticut, 2008. *J Community Health* 2010: 35(5): 495-499.

14. Quirós S, de la Rosa D, Uranga A, Madero R, et al. Screening for Latent Tuberculosis Infection in Patients who are Candidate for Biological Therapies in Spain? A Multidisciplinary Survey. *Arch Bronconeumol* 2018: 54(10): 510-517.

15. Salazar-Schicchi J, Jedlovsky V, Ajayi A, Colson PW, Hirsch-Moverman Y, El-Sadr W. Physician attitudes regarding bacille Calmette-Guerin vaccination and treatment of latent tuberculosis infection. *Int J Tuberc Lung Dis* 2004: 8(12): 1443-1447.

16. Smith MY, Attig B, McNamee L, Eagle T. Tuberculosis screening in prescribers of anti-tumor necrosis factor therapy in the European Union. *Int J Tuberc Lung Dis* 2012: 16(9): 1168-1173.

17. Stout JE, Ostbye T, Walter EB, Hamilton CD. Tuberculosis knowledge and attitudes among physicians who treat young children in North Carolina, USA. *Int J Tuberc Lung Dis* 2006: 10(7): 783-788.

18. Tran NQ, Garcia-Rosell M, Pattanaik D, Raza SH, Carbone L. Screening and Treatment of Latent Tuberculosis Among Patients Receiving Biologic Agents: A National and International Survey of Rheumatologists. *J Clin Rheumatol* 2017: 23(1): 6-11.

19. Vinnard C, Linkin D, Behrman A. Current Approach to Latent Tuberculosis Diagnosis and Treatment among Medical Center Occupational Health Physicians. *Infect Control Hosp Epidemiol* 2012: 33(12): 1262-1265.

20. Xerinda S, Oliveira O, Lucas R, et al. Gaps and barriers to tuberculosis screening among anti-tumor necrosis factor prescribers. *Acta Reumatol Port* 2016: 41(4): 382-384.

21. Yates F, Janakiraman A, Headly A, Linkin DR, Vinnard C. Attitudes Towards Latent Tuberculosis Among Physicians in Training: The Role of BCG Vaccination. *J Community Health* 2015: 40(2): 364-366.

22. Chiang SS, Cruz AT, Del Castillo H, Contreras C, Becerra MC, Lecca L. Evaluation of health-care providers' knowledge of childhood tuberculosis in Lima, Peru. *Paediatr Int Child Health* 2015: 35(1): 29-35.

23. Mirtskhulava V, Whitaker JA, Kipiani M, et al. Determinants of Tuberculosis Infection Control-Related Behaviors Among Healthcare Workers in the Country of Georgia. *Infect Control Hosp Epidemiol* 2015: 36(5): 522-528.

24. Moolphate S, Lawpoolsri S, Pungrassami P, Sanguanwongse N, Yamada N, Kaewkungwal J. Barriers to and motivations for the implementation of a treatment programme for latent tuberculosis infection using isoniazid for people living with HIV, in upper northern Thailand. *Global J Health Sci* 2013: 5(4): 60-70.

25. Pathak V, Harrington Z, Dobler CC. Attitudes towards preventive tuberculosis treatment among hospital staff. *PeerJ* 2016.

26. Ramos J, Wakoff-Pereira MF, Cordeiro-Santos M, et al. Knowledge and perceptions of tuberculosis transmission and prevention among physicians and nurses in three Brazilian capitals with high incidence of tuberculosis. *J Bras Pneumol* 2018: 44(2): 168-170.

27. Spruijt I, Haile DT, Erkens C, et al. Strategies to reach and motivate migrant communities at high risk for TB to participate in a latent tuberculosis infection screening program: a community-engaged, mixed methods study among Eritreans. *BMC Public Health* 2020: 20(1).

28. Alotaibi B, Yassin Y, Mushi A, et al. Tuberculosis knowledge, attitude and practice among healthcare workers during the 2016 Hajj. *PloS one* 2019: 14(1): e0210913.

29. Trajman A, Wakoff-Pereira MF, Ramos-Silva J, et al. Knowledge, attitudes and practices on tuberculosis transmission and prevention among auxiliary healthcare professionals in three Brazilian high-burden cities: a cross-sectional survey. *BMC Health Serv Res* 2019: 19..

30. Coreil J, Lauzardo M, Heurtelou M. Anticipated Tuberculosis Stigma among Health Professionals and Haitian Patients in South Florida. *J Health Care Poor Underserved* 2012: 23(2): 636-650.

31. Hirsch-Moverman Y, Wall K, Weinfurter P, et al. Acceptability of interferon-gamma release assays among healthcare workers who receive routine employee tuberculosis testing. *Int J Occup Environ Health* 2013: 19(4): 319-324.

32. Jackson M, Harrity S, Hoffman H, Catanzaro A. A survey of health professions students for knowledge, attitudes, and confidence about tuberculosis, 2005. *BMC Public Health* 2007: 7.

33. *Infect Control Hosp Epidemiol* 2015: 36(5): 522-528.

46. Montagna MT, Mascipinto S, Pousis C, et al. Knowledge, experiences, and attitudes toward Mantoux test among medical and health professional students in Italy: a cross-sectional study. *Ann Ig* 2018: 30(5): 86-98.

34. Montagna MT, Napoli C, Tafuri S, et al. Knowledge about tuberculosis among undergraduate health care students in 15 Italian universities: a cross-sectional study. *BMC Public Health* 2014: 14(1): 970.

35. Zhou F, Zhang L, Gao L, et al. Latent tuberculosis infection and occupational protection among health care workers in two types of public hospitals in China. *PloS one* 2014: 9(8): e104673..

36. Ailinger RL, Armstrong R, Nguyen N, Lasus H. Latino immigrants' knowledge of tuberculosis. *Public Health Nurs* 2004: 21(6): 519-523.

37. Biedenharn AM. Assessment of Knowledge and Attitudes on Latent Tuberculosis Treatment Acceptance in a Southwest Ohio Public Health Clinic. 2015. Wright State University, Dayton, Ohio.

38. Butcher K, Biggs B-A, Leder K, Lemoh C, O'Brien D, Marshall C. Understanding of latent tuberculosis, its treatment and treatment side effects in immigrant and refugee patients. *BMC Res Notes* 2013: 6: 342-342.

39. Colson PW, Franks J, Sondengam R, Hirsch-Moverman Y, El-Sadr W. Tuberculosis Knowledge, Attitudes, and Beliefs in Foreign-born and US-born Patients with Latent Tuberculosis Infection. *J Immigr Minor Health* 2010: 12(6): 859-866.

40. Colson PW, Hirsch-Moverman Y, Bethel J, et al. Acceptance of treatment for latent tuberculosis infection: prospective cohort study in the United States and Canada. *Int J Tuberc Lung Dis* 2013: 17(4): 473-479.

41. DeLuca A, Dhumal G, Paradkar M, et al. Addressing knowledge gaps and prevention for tuberculosis-infected Indian adults: a vital part of elimination. *BMC Infect Dis* 2018: 18(1): 202.

42. Gao J, Berry NS, Taylor D, Venners SA, Cook VJ, Mayhew M. Knowledge and Perceptions of Latent Tuberculosis Infection among Chinese Immigrants in a Canadian Urban Centre. *Int J Family Med* 2015: 2015.

43. Hill L, Blumberg E, Sipan C, et al. Multi-Level Barriers to LTBI Treatment: A Research Note. *J Immigr Minor Health* 2010: 12(4): 544-550.

44. Kane M, Korn B, Saukkonen J, et al. Barriers to accepting and completing latent tuberculosis infection treatment. *Ir Med J* 2013: 106(7): 200-204.

45. Li Y, Zheng YH, Lu LP, et al. Acceptance of Chemo-prophylaxis for Latent Tuberculosis Infection among High School/College Student Contacts of Tuberculosis Patients in Shanghai, China. *Biomed Environ Sci* 2018: 31(4): 317-321.

46. O'Donnell MR, Coe A, Bliss C, et al. Acceptance of interferon-gamma release assay by a high-risk urban cohort. *Int J Tuberc Lung Dis* 2011: 15(10): 1334-1339.

47. Narayanan N, Gulati N, Ghoshal B, et al. Latent Tuberculosis Infection Beliefs and Testing and Treatment Health Behaviors Amongst Non-US-Born South Asians in New Jersey: A Cross-Sectional Community Survey. *J Community Health* 2019: 44(4): 796-804.

48. Walker CL, Duffield K, Kaur H, Dedicoat M, Gajraj R. Acceptability of latent tuberculosis testing of migrants in a college environment in England. *Public Health* 2018: 158: 55-60.

49. Howley MM, Rouse CD, Katz DJ, Colson PW, Hirsch-Moverman Y, Royce RA, Tuberculosis Epidemiologic Studies C. Knowledge and Attitudes About Tuberculosis Among U.S.-Born Blacks and Whites with Tuberculosis. *J Immigr Minor Health* 2015: 17(5): 1487-1495.

50. Skinner D, Mandalakas AM. Pasting together the preventive therapy puzzle. *Int J Tuberc Lung Dis* 2013: 17(2): 175-177.
